# Niche differentiation among annually recurrent coastal Marine Group II Euryarchaeota

**DOI:** 10.1038/s41396-019-0491-z

**Published:** 2019-08-26

**Authors:** Luis H. Orellana, T. Ben Francis, Karen Krüger, Hanno Teeling, Marie-Caroline Müller, Bernhard M. Fuchs, Konstantinos T. Konstantinidis, Rudolf I. Amann

**Affiliations:** 1 Department of Molecular Ecology, Max Planck Institute for Marine Microbiology, Bremen, D-28359 Germany; 2 School of Civil and Environmental Engineering, Georgia Institute of Technology, Ford Environmental Science and Technology Building, 311 Ferst Drive, Atlanta, GA 30332 USA

**Keywords:** Water microbiology, Molecular ecology, Metagenomics

## Abstract

Since the discovery of archaeoplankton in 1992, the euryarchaeotal Marine Group II (MGII) remains uncultured and less understood than other planktonic archaea. We characterized the seasonal dynamics of MGII populations in the southern North Sea on a genomic and microscopic level over the course of four years. We recovered 34 metagenome-assembled genomes (MAGs) of MGIIa and MGIIb that corroborated proteorhodopsin-based photoheterotrophic lifestyles. However, MGIIa and MGIIb MAG genome sizes differed considerably (~1.9 vs. ~1.4 Mbp), as did their transporter, peptidase, flagella and sulfate assimilation gene repertoires. MGIIb populations were characteristic of winter samples, whereas MGIIa accounted for up to 23% of the community at the beginning of summer. Both clades consisted of annually recurring, sequence-discrete populations with low intra-population sequence diversity. Oligotyping of filtered cell-size fractions and microscopy consistently suggested that MGII cells were predominantly free-living. Cells were coccoid and ~0.7 µm in diameter, likely resulting in grazing avoidance. Based on multiple lines of evidence, we propose distinct niche adaptations of MGIIa and MGIIb *Euryarchaeota* populations that are characteristic of summer and winter conditions in the coastal North Sea.

## Introduction

Since the discovery of marine planktonic archaea using culture-independent approaches [1, 2], four major archaeal lineages have been characterized in marine ecosystems. The thaumarchaeotal Marine Group I (MGI) and the euryarchaeotal Marine Group II (MGII) have been found in deep and surface ocean waters, respectively [3–5]. In addition, archaea belonging to the euryarchaeotal Marine Group III (MGIII) [6, 7] and IV (MGIV) [8] have been mostly associated with deep sea environments. So far, only members of the MGI group have been isolated [9]. Thus, the characterization of other marine archaea has been limited to culture-independent approaches. MGI have been mostly associated with ammonia oxidation [9], whereas less is known about MGII. Nonetheless, sequences recovered from metagenomes belonging to the MGIIa subgroup have suggested a potential role in protein and fatty acid degradation [10–12]. It has been proposed that MGII could thrive under oligotrophic conditions, where particle-associated MGII can outnumber free-living cells of the same group [13]. In addition, a partially recovered genome of the MGIIb group (originally proposed as class ‘*Thalassoarchaea*’), obtained from the Mediterranean deep chlorophyll maximum, showed similar heterotrophic features as those found in MGIIa genomes, but have a lower GC percentage and a smaller genome size [14]. Hence the high diversity recently reported [11, 12] within MGII populations underscores the knowledge gaps for these marine archaeal clades.

Our understanding of the dynamics of MGII populations has until recently [11, 12] been restricted to the use of molecular markers such as 16S rRNA gene sequences [15–17]. Studies in the Mediterranean have suggested niche separation between MGI and MGII, but also within MGII populations. For instance, while MGIIb (and MGI) dominated at the time of winter sampling, MGIIa were abundant during summer. These seasonal differences were proposed to result from strong changes in biotic (e.g., nutrient and phytoplankton stocks) and abiotic factors (e.g., temperature and water column stratification) over the winter-summer transition [15]. Previous reports have indicated short blooms of MGII populations during summer [3, 15, 18] following phytoplankton bloom events and also coinciding with decreased levels of chlorophyll *a* [17, 19]. Phytoplankton blooms in coastal marine environments often cause successions of bacterioplankton communities participating in the remineralization of the released organic matter [20], which are dominated by members of the classes *Flavobacteriia*, *Alphaproteobacteria* and *Gammaproteobacteria* [21–23]. Still, the diversity of MGII populations occurring before and after these blooms have received much less attention due to their usually lower abundance compared to heterotrophic bacterioplankton [17, 24]. A recent large phylogenomic comparison of MGII flagellum-based adhesion, transport and degradative potential suggests that multiple MGII clades diversified from a surface water-dwelling photoheterotrophic ancestor [11]. However, little is known about how the genomic potential of distinct MGII clades correlates with observed temporal abundance patterns. In addition, not much is known about the level of genetic heterogeneity within recurrent MGII populations, which is important for delimiting MGII ecophysiological niches including ecotypes and their impact on coastal environments.

We combined fluorescence in situ hybridization (FISH) and metagenome sequencing approaches to explore the temporal patterns and metabolic potentials of discrete euryarchaeotal populations in the southern North Sea (German Bight) in the years 2009 to 2012. The recovery of 34 high-quality metagenome-assembled genomes (MAGs) related to MGII allowed us to compare the genomes and ecology of MGIIa and MGIIb. Our results reveal low intra-population diversity of annually recurrent MGII populations and provides novel information on the potential niche differentiation of summer and winter clades of photoheterotrophic Marine Group II *Euryarchaeota*.

## Materials and methods

### Sampling and sequencing

Surface seawater samples were collected from the ‘Kabeltonne’ ecological research site off the North Sea island of Helgoland (54^◦^ 11.3′ N, 7^◦^ 54.0′ E) as described previously [23, 25, 26]. Briefly, samples for DNA sequencing were passed through 10 and 3 µm pore-size filters before cells were collected on 0.2 µm pore-size polycarbonate filters. This approach removed phytoplankton and most of the particle-associated microorganisms. Seawater for cell counting was also collected from the surface, but it was not fractionated [23, 26]. The sequencing of 44 surface water metagenomes from ‘Kabeltonne’ was performed at the DOE Joint Genome Institute (DOE-JGI) as described previously [25, 26]. Metagenomes from 2009 [23] were sequenced on the GS FLX Ti platform (454 Life Sciences, Branfort, CT, USA), all others on the Illumina HiSeq platform (Illumina, San Diego, CA, USA) with paired-end sequencing [25]. Trimming and processing of raw reads was performed as previously published [26].

### Catalyzed reporter deposition fluorescence in situ hybridization

For the enumeration of *Euryarchaeota*, 100 ml of water were fixed with 1% formaldehyde at room temperature for 1 h, filtered onto 47 mm diameter polycarbonate filters (pore size 0.2 µm) with a vacuum of at most 100 mbar. Catalyzed Reporter Deposition Fluorescence in situ hybridization (CARD-FISH) was performed as outlined before [27]. A detailed protocol is available in the supplementary text.

### MAG recovery

Archaeal MAGs were obtained from previously assembled 2010–2012 Helgoland metagenomes [26]. Briefly, de novo assembly of short-read metagenomes was performed using SPADES 3.10 [28] and assembled contigs longer than 2.5 kbp were binned using CONCOCT [29] as part of the standard Anvi’o v3 metagenomic workflow [30]. Differential coverage information was generated by mapping reads from four additional randomly selected dates from the same year. Reads were mapped using BBMap v35.14 (http://bbtools.jgi.doe.gov; ‘fast’ mode, minid = 0.99, and idfilter = 0.97). Manual inspection for congruent genetic composition and coverage of recovered MAGs was performed in Anvi’o [30]. Selected MAGs (%[completion]−4 × % [contamination] ≥ 50 (determined by checkM [31])) were de-replicated using dRep v2.2.3 [32], which combines MASH and average nucleotide identity (ANI > 95%) measures in order to select the best representative from each group of highly similar MAGs (Table S1). Genes were predicted using Prodigal v2.6.3 [33] (metagenome option). Reported taxonomic classification, completion and contamination for archaeal MAGs was determined using the Microbial Genome Atlas webserver (MiGA) [34]. MAGs were uploaded to MiGA using the “popgenome” dataset option. MiGA determines completeness and contamination using previously reported single-copy marker genes [35]. Taxonomic classifications are based on genome-aggregate average amino acid and average nucleotide identity (AAI/ANI) compared to available reference genomes. Additional details about genomic comparisons and gene annotation are available in the supplementary text.

MGII MAGs previously reported [11, 12] were also included in the phylogenetic reconstructions. Identical and redundant MAGs based on ANI were de-replicated using FastANI [36] and quality (%[completion]−4 × %[contamination] ≥ 50). Briefly, using an ANI value ≥99% reduced the MGII MAG collection [11, 12] from 643 to 294 de-replicated MAGs. Representative MAGs for each 99% ANI group were selected based on highest completion and lowest contamination (Table S2 and S3).

### MAG abundances and intra-population sequence diversity

Bowtie2 [37] was used to map short-reads to MAG contigs, and SamTools [38] to convert the resulting SAM files to BAM files. Genomecov (-bga option) from the Bedtools package [39] was used to determine sequencing depth. In order to exclude biases introduced by highly conserved regions and contig edges, the 80% central truncated average of the sequencing depth (TAD) of all bases was determined using the ‘BedGraph.tad.rb’ script (option range 80) from the enveomics collection [35]. The option ‘range 80’ removes the top 10% and bottom 10% positions of per base sequencing coverage in each MAG. The *rpoB* sequencing depth was used as a proxy for the sequencing depth for all microbial genomes in each metagenome assuming that both whole genome and *rpoB* sequencing depths were equal. The sequencing depths of *rpoB* genes was determined for each metagenome by first identifying short-reads related to *rpoB* genes using ROCker [40]. Briefly, a custom database previously constructed for RpoB sequences [41] was used in BLASTx searches [42] for each metagenome. BLASTx outputs were then filtered for high-quality matches using a ROCker model specific to the RpoB database. Finally, MAG abundance values were calculated as the quotient between the determined TAD for each MAG and the sequencing depth of its *rpoB* gene.

Intra-population sequence diversity was determined as previously reported [43], by first mapping the short-reads from different metagenomes to representative MAGs using BLASTn (-outfmt 6 option). Samples from different time points where individual MAGs represented >0.01%—corresponding to ~2× coverage or more of the total population—were selected. Read recruitment plots were generated using the BLASTn outputs and processed with the “BlastTab.catsbj.pl” and “enve.recplot2” scripts from the enveomics collection for each MAG and sample [35]. The nucleotide identity value denoting the sequence discontinuity for mapped reads was selected upon visual inspection as previously described [44], identifiable as a drop in coverage by 3–4 orders of magnitude (see example in Fig. S5). For all *Euryarchaeota* MAGs, 98% identity represented the cutoff for sequence-discrete populations. Reads mapping with identities above this threshold based on BLASTn searches were thus used to calculate read-based average nucleotide identity (ANIr), as a proxy for intra-populations diversity using the “enve.recplot2.ANIr” R script from the enveomics collection.

### MAG and gene phylogenies

MAG phylogenetic analyses were performed using 15 syntenic ribosomal proteins (L2, L3, L4, L5, L6, L14, L15, L18, L22, L24 and S3, S8, S10, S17, S19) [45–47]. Predicted protein sequences were detected in each MAG using target hidden Markov models (HMMs) for each protein obtained from TIGRFAM [48], AMPHORA2 [49], and Pfam [50] using HMMER v3.2.1 [51]. Multiple alignments for each protein were generated using ClustalΩ [52] and a concatenated alignment was generated using the ‘Aln.cat.rb’ script [35]. Maximum-likelihood phylogenetic estimations were determined in RAxML v8.0.19 [53] (PROTGAMMAAUTO, -N 500) and visualized in the interactive Tree of Life (iTol) [54].

### 16S rRNA gene oligotyping

Sample collection at Helgoland and processing for amplicon analysis has been described previously [19]. Briefly, the two fractions corresponding to size ranges of 0.2 to 3 µm, and 3 to 10 µm were separated via filtration of the surface water samples (*~*1 m depth). Amplicons were generated for both fractions via PCR amplification of the V4 region of the 16S rRNA gene and sequenced using Illumina MiSeq 2 × 250 bp chemistry at the DOE-JGI. Differentially abundant MGII oligotypes were determined with the DESeq2 package [55]. Additional details and complete methods can be found in the supplementary material.

## Data availability

Metagenomes were previously deposited in NCBI (see Table S1 for BioProject accession numbers). Helgoland MAGs used in this study were previously deposited at the European Nucleotide Archive (Study PRJEB28156) using the data brokerage service of the German Federation for Biological Data (GFBio) [56]. Other MGII MAGs published were obtained using the accession numbers provided [11, 12]. Raw 16S rRNA gene amplicon data are stored by JGI in the GOLD database under the project IDs Gp0056779 (‘free living’; from 0.2 to 3 µm), and Gp0072732 and Gp0072733 (‘attached’; from 3 to 10 µm), as part of the community sequencing project COGITO.

## Results

### Metagenome-assembled genomes from Helgoland

**Fig. 1:**
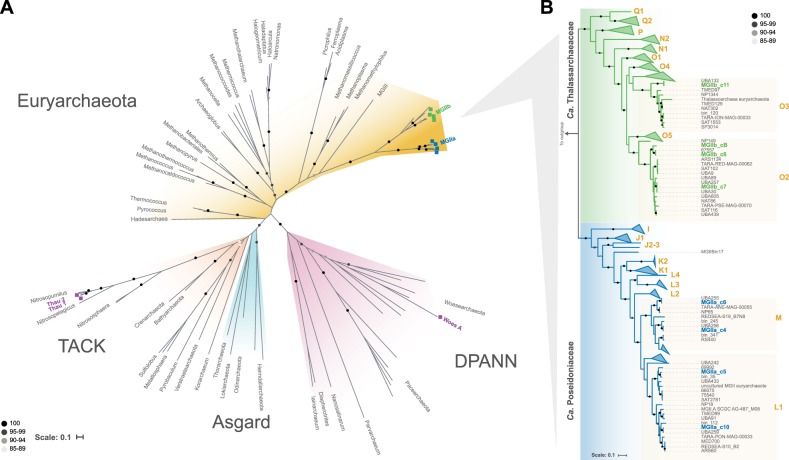
Phylogenetic reconstruction of archaeal genomes including MAGs from Helgoland. **a** The reconstruction of the maximum likelihood phylogeny used representative genomes of DPANN, TACK, *Euryarchaeota*, and Asgard groups and was based on a syntenic block of 15 conserved ribosomal archaeal genes (see methods). Colored squares represent recovered MAGs in this study. Main proposed genera names are displayed. Branch support values between 85–100% are represented by dots. **b** Phylogenetic reconstruction of MGII MAGs from Helgoland and others recovered from elsewhere around the world [11, 12]. Clades were collapsed according to genera previously reported [11]. Clades L1, M, O2, and O3 were not collapsed to highlight the phylogenetic relationship of Helgoland MAGs to other previously reported MGII MAGs

During the spring phytoplankton blooms of 2009–2012, we collected water samples at the ‘Kabeltonne’ station, Helgoland Island. We have previously reported an extensive time-series of the characteristics of this shallow (water depth ~10 m) coastal marine site and the sampling strategies [25]. Given that all metagenomes from the time-series were assembled and binned separately, many highly similar MAGs were obtained (see ANI values between MGII MAGs in Fig. S1). MAGs classified as archaeal were reduced from a total of 49 down to 11 representative MAGs using an ANI threshold of 95% and quality over 50 (see Methods) (Table 1, S1). The reconstruction of the MAG phylogeny using a syntenic block of conserved ribosomal genes [45–47] provided an overview of the archaeal diversity at Helgoland (Fig. 1a). Eight of the representative MAGs belonged to the *Euryarchaeota* phylum, two to the TACK superphylum, and one to the DPANN group (Fig. 1a). The topology of a phylogenetic tree using recovered 16S rRNA gene sequences from some of the MAGs (5/11; see Table S1) was congruent with the tree using conserved ribosomal genes (Fig. S2).

**Table 1:** Genome statistics for eleven representative archaeal MAGs

MAG	Phylum	MAG length (Mbp)	Number of contigs	Number of predicted genes	GC content (%)	Completeness (%)	Contamination (%)	Sampling date
MGIIa_c4	Euryarchaeota	1.79	43	1511	51.9	88.5	3.8	08-Mar-2012
MGIIa_c5		1.93	48	1631	51.5	96.2	0	31-May-2012
MGIIa_c6		1.98	38	1652	50.1	96.2	0	26-May-2011
MGIIa_c10		1.96	49	1619	49.3	96.2	0	24-May-2012
MGIIb_c7		1.39	73	1267	38.2	92.3	0	08-Mar-2012
MGIIb_c8		1.41	31	1244	38.6	88.5	0	21-Mar-2011
MGIIb_c11		1.62	25	1448	36.8	96.2	0	28-Apr-2011
MGIIb_cB		1.33	187	1231	37.4	80.8	0	24-Mar-2011
Thau_1	TACK	1.14	86	1483	30.9	88.5	0	21-Mar-2011
Thau_2		1.07	82	1404	31.3	92.3	0	03-Mar-2010
Woes_A	DPANN	0.71	158	869	36.0	50	0	04-May-2010

The two thaumarchaeotal MAGs (TACK superphylum), Thau1 and Thau2, were placed within the MGI.1a clade and shared 88.7% AAI (SD 9.8%, 1207 protein sequences) and probably represent distinct species of the same genus. Their genome sizes (~1.1 Mbp) and numbers of predicted genes (~1400) were similar. Thau1 and Thau2 shared 88.9 and 95.7% ANI with *Ca*. Nitrosomarinus catalina, respectively. This MGI archaeon, possibly representing the same species as Thau2, was isolated from the San Pedro Ocean time-series (temperate Pacific Ocean waters) off the coast of California [57]. The DPANN MAG was placed within the *Woesearchaeota*, but shared only ~40% AAI with previously reported *Woesearchaeota* MAGs.

The eight *Euryarchaeota* MAGs were divergent from the available reference archaeal genomes and only shared AAI values between ~47 to 80% to the genome of an uncultured MGII euryarchaeote [10] when compared to 11,568 classified reference genomes and isolates [34] (Table S1). Nonetheless, higher AAI values for Helgoland MAGs were obtained (~71.5 and ~79.3% for MGIIa and MGIIb, respectively) when compared to previously recovered MGII MAGs of the highlighted clades on Fig. 1b. The completion for all *Euryarchaeota* MAGs was high, with values up to 96.2% and low levels of contamination (Table 1). Interestingly, the *Euryarchaeota* genomes were separated into two distinct clades, previously described as MGIIa and MGIIb [11, 12, 14, 16, 58]. Similar phylogenetic relationships were obtained using a conserved syntenic block of ribosomal proteins and 120 single-copy markers previously described for MGII genomes [11, 59] (Fig. S3a, b). AAI among MGIIa MAGs ranged between 64–73 and 63–85% for MGIIb MAGs (Fig. S4). Larger average genome sizes (1.9 vs. 1.4 Mbp), and higher %GC values (50.5 vs. 37.5%) were characteristic of MAGs from MGIIa compared to MGIIb, respectively, in agreement with previous findings [11, 12].

A recent study using rank-normalized phylogeny [11] proposed the order level *Ca*. Poseidoniales for the MGII lineage and the subgroups MGIIa and MGIIb as *Ca*. Poseidoniaceae and *Ca*. Thalassarchaeaceae families, respectively, in addition to 21 new genera within these families. According to this classification, the Helgoland MGIIa MAGs belong to the *Ca*. Poseidoniaceae family and the genera L1 (MGIIa_c5 and MGIIa_c10) and M (MGIIa_c4 and MGIIa_c6). MGIIb MAGs are members of the *Ca*. Thalassarchaeaceae family in the genera O3 (MGIIb_c11) and O2 (MGIIb_c8, MGIIb_cB, and MGIIb_c7). MGIIa_c6 corresponds to the proposed type species *Ca*. Poseidonia alphae as both MAGs share 99.98% ANI and were recovered from the same metagenome. Thus, these two MAGs likely represent the same population yet both MAGs are not identical since they were recovered using different assembly and binning strategies.

### Abundance and intra-population sequence diversity dynamics of MGII MAGs

**Fig. 2:**
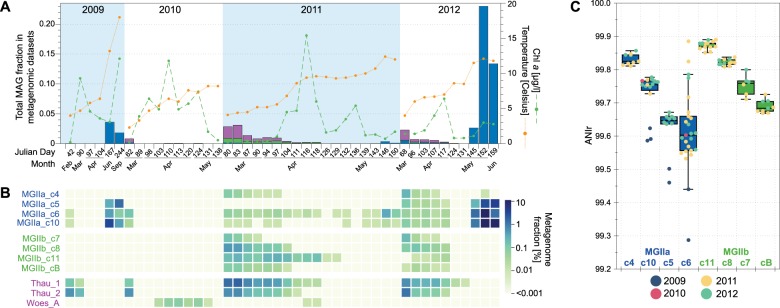
Abundance of MAGs during coastal spring algae blooms at Helgoland in 2011 and 2012. **a** Fraction of main archaeal groups in each spring bloom metagenome. Chlorophyll *a* and temperature measurements of the same samples are also displayed. **b** Individual abundance of each MAG (measured as fraction of the metagenome). Note that separation between samples does not represent a continuous time scale on the *x*-axis. **c** Average nucleotide identity for reads mapping *Euryarchaeota* MAGs (ANIr) representing >0.01% of the microbial communities (~2×) in 2009–2012 metagenomes

The metagenome time-series allowed us to evaluate the abundance and persistence of the recovered MAGs over the course of the 2009–2012 spring blooms. Previous studies have focused on the rapid succession patterns of the *Flavobacteriia*, *Gammaproteobacteria*, and *Roseobacter* bacterial classes [23, 25]. The abundances of the recovered archaeal MAGs were low for most of the samples (Fig. 2a, b). Nonetheless, MGII MAGs were recurrent and most abundant in late winter and early summer, before and after the phytoplankton blooms. Specifically, MGI and MGIIb MAGs were detected in pre-bloom metagenomes, whereas MGIIa were dominant in post-bloom metagenomes in two of the four years. The MGIIa_c6 MAG represented the most prevalent population as it was detected in 90% of the post-bloom metagenomes (9/10) and 65.7% of all metagenomes (25/38). Metagenomes obtained during 2010 were mostly obtained during the spring phytoplankton bloom, therefore MGII populations were almost undetectable in the samples from this year (Fig. 2a, b). MGIIb MAGs were only detected in samples obtained during 2011–2012, with MGIIb_c11 being most prevalent.

In pre-bloom metagenomes from 2011 and 2012, MGIIa and MGIIb MAGs comprised, on average, 0.1 and 0.3% of the total microbial fraction, respectively. Notably, MAGs affiliated to the MGIIa populations comprised up to 23% of the total microbial community in post-bloom metagenomes during 2012. In fact, individually, MGIIa_c6 and MGIIa_c10 comprised up to ~10% of the total microbial community in post-bloom metagenomes of 2012. The exception to the pattern described above for MGIIa MAGs was the MGIIa_c4 MAG, which had an abundance distribution similar to MGIIb populations (i.e., mostly detected during winter) despite both MGIIa_c4 and MGIIa_c6 MAGs belong to the same genus (Fig. 1b).

We analyzed the intra-population sequence diversity of discrete populations by mapping short-metagenomic reads from metagenomes to the MGII MAGs and determining ANIr values. This approach allows a quantitative evaluation of sequence diversity for sequence-discrete populations and is less prone to systematic issues carried by missassemblies or differences in the degree of completion when comparing recovered MAGs. In addition, the use of MAGs as reference sequences in recruitment plots that were assembled from single metagenomes allowed us to preserve the genetic characteristics of discrete archaeal populations at each sampling point (e.g., recruitment plot for MGIIa_c6 in Fig. S5). The ANIr values ranged from 99.2 to 99.8%, indicating a low intra-population sequence diversity (or highly clonal, Fig. 2c). Even though the variation in ANIr values among metagenomes (when the same reference genome was used in the recruitment of reads from different metagenomes) was small for both MGII populations, the interquartile range of ANIr values for MGIIa was higher compared to MGIIb MAGs (two tailed *t*-test, *P* *<* 0.01), indicating a higher intra-population sequence variability for MGIIa populations. For instance, the interquartile range of ANIr values for MGIIa_c6 was the highest (IQR = 0.6) among MGII MAGs.

### Visualization and quantification of MGII abundance

**Fig. 3:**
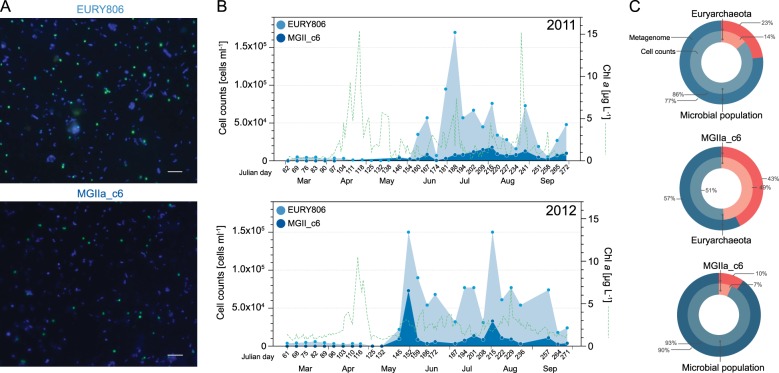
Cell morphology and in-situ abundances of MGIIa_c6 populations at Helgoland based on microscopy. **a** CARD-FISH results for sample from 31-May-2012. DAPI and MGIIa_c6 probe. The scale represents 5 µm. **b** Counts of total *Euryarchaeota* (light blue) and MGIIa_c6 (blue) cells using the EURY806 and MGIIa_c6 probes, respectively. **c** Comparison of relative abundances obtained from metagenome (outer circle) and cell counts (inner circle) approaches for *Euryarchaeota* from 31-May 2012 with respect to the total microbial population (top), MGIIa_c6 with respect to *Euryarchaeota* (middle) and total microbial population (bottom)

We visualized and quantified marine *Euryarchaeota* populations using FISH and CARD-FISH. The CARD-FISH approach allowed us to confirm and expand on the temporal distribution patterns of archaeal populations observed in the metagenomic data. In addition to quantifying all MGII *Euryarchaeota* with class-specific oligonucleotide probe EURY806 [60], we chose to follow clade MGIIa_c6 because it was among the abundant populations that also contained a 16S rRNA gene sequence for probe design (Table S1). The vast majority of the cells detected using probe EURY806 and MGIIa_c6 were coccoid, with a diameter of <1 µm, suggesting that MGIIa and MGIIb had similar cell morphologies. The cells were neither accumulated in aggregates nor attached to particles, and appeared to be mostly planktonic (Fig. 3a and S6). The average cell diameter for MGIIa_c6 cell was 0.7 ± 0.2 µm. *Euryarchaeota* represented a relatively small fraction of the microbial community before and during the phytoplankton spring blooms (i.e., from March to May), but dominated by the end of May and the beginning of June of 2011 and 2012, respectively (Fig. 3b and S7).

The relative abundance patterns observed for *Euryarchaeota* and MGIIa_c6 populations using CARD-FISH were consistent with the abundances determined in metagenomes. For instance, on May 31st of 2012 (Julian day 152), *Euryarchaeota* peaked in abundance, accounting for 23 and 14% of the microbial populations in metagenomes and cell counts, respectively (Fig. 3c, top circle). Similarly, MGIIa_c6 cells with respect to euryarchaeotal cells accounted for 43, 49, and 10% on May 24th, 31st and June 6th of 2012 (145, 152, and 159 Julian days) while at the metagenome level the corresponding values were 52, 43, and 9% for the same dates (Julian day 152 showed in Fig. 3c, middle circle). Interestingly, MGIIa_c6 represented, as a single species-level clade, 7 and 10% of visualized cells and metagenome, respectively (Fig. 3c, bottom circle).

### Oligotyping of size fractions

**Fig. 4:**
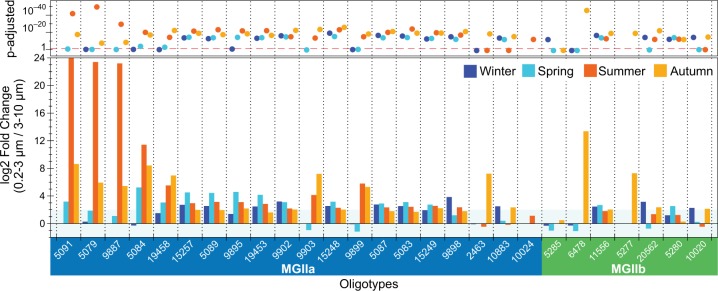
Distribution of MGII oligotypes in Helgoland surface waters. Differential abundance of MGII oligotypes was performed between 0.2–3 µm and 3–10 µm pore-size fractions within seasons in DESeq2. The lower panel shows the log2-fold values between 0.2–3 µm (values above zero) and 3–10 µm (values below zero) pore-size fractions for each of the 27 oligotypes. Oligotypes were selected based on relative abundance of at least 0.1% in at least one sample. The top panel shows the *p*-adjusted values determined for the comparison between size fractions and the dashed line demarcates the *p*-adjusted = 0.05 value

The examination of MGII 16S rRNA gene oligotypes obtained from 0.2–3 and 3–10 µm pore-size fractions allowed us to further investigate the association of MGII populations with particles in an expanded time scale. A total of 27 recurrent MGII oligotypes, of which 21 corresponded to MGIIa and 6 to MGIIb, had above 0.1% abundance in at least one sample from 2010, 2011, and 2012 (Fig. S8). MGIIa oligotypes were more abundant during summer/autumn, whereas MGIIb were mostly detected in winter/autumn, in agreement with our metagenomic results. Interestingly, 59 to 89% of the MGII oligotypes were 4-fold higher in the 0.2–3 µm fraction in all seasons (log2-fold > 2; Fig. 4). Although 9 MGII oligotypes were slightly enriched in the 3–10 µm fraction mostly during spring, they were never above 2-fold higher (log2-fold < −1), indicating a higher prevalence of MGII populations in the smaller 0.2–3 µm pore-size fractions.

### Metabolic potential of North Sea Euryarchaeota

Core metabolic functions indicating a heterotrophic lifestyle, such as the capacity to perform glycolysis, and the citric acid cycle were detected in all MGII MAGs from Helgoland, and are consistent with previous analyses based on globally recovered MGII MAGs [11, 12, 58]. Nonetheless, differences such as the absence or reduced number of genes for key metabolic functions were noted among MGII MAGs from Helgoland. For instance, while MGIIa MAGs encode all necessary enzymes for non-oxidative pentose formation, MGIIb MAGs from Helgoland of O2 genera (MGIIb_c7, c8, and cB) lack the ribose-phosphate pyrophosphokinase gene (*prs*), necessary for the phosphoribosyl pyrophosphate (PRPP) and de novo generation of purine nucleotides and several amino acids. The lack of the *prs* gene was also consistent in the majority of the representative MGII O2 MAGs used for the phylogenetic reconstruction (3/17) but not in the O3 genus (9/10), where this feature was prevalent. In addition, different loci containing the genes *flaB*, *flaH*, *flaI*, and *flaJ*, which commonly form part of the flagellar structure [61], were present in all MGIIa MAGs. In contrast, only MGIIb_c11 among MGIIb MAGs contained a locus encoding these flagellar genes (Table S4). Helgoland MGIIa MAGs possessed additional components of the flagella machinery including the *flaJ*, the ATPases *flaI* and *tadC* genes. However, no known chemotaxis components were detected in any of the Helgoland MGII MAGs, in agreement with previous findings [11].

**Fig. 5:**
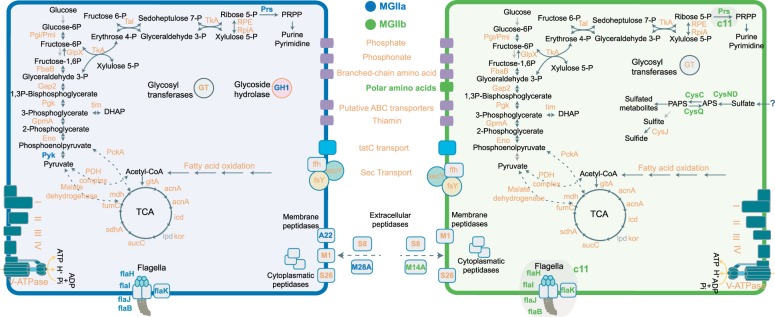
Graphical representation of the predicted metabolic capabilities of MGII MAGs. Functions exclusively detected in MGIIa and MGIIb MAGs are colored in blue and green, respectively. Predicted functions shared by the two groups are colored in magenta. Substrates and products from depicted pathways are not necessarily balanced. A more detailed gene description is available in Tables S4, S5, and S6

Unlike heterotrophic bacteria linked to phytoplankton blooms, a low number of glycoside hydrolases (GH) were detected in MGII MAGs. In fact, only a single glycoside hydrolase belonging to family 1 (GH1) was detected in MGIIa MAGs (MGIIa_c4, MGIIa_c5, and MGIIa_c10) and no previously described GHs were found in MGIIb MAGs. The GH1 belongs to the wide-spread family of beta-glucosidase enzymes (EC 3.2.1.-), involved in the processing of polysaccharides and oligosaccharides. In contrast, both MGII groups encoded several copies of glycosyltransferases (GT) involved in the formation of glycoside linkages, among other predicted functions (Table S4). A more detailed description of the metabolic potential (Fig. 5) and predicted protein orthologs (Fig. S9) is available in the supplementary text.

### Potential for assimilatory sulfate reduction in MGIIb MAGs

Another difference between the two family-level MGII groups in Helgoland was the potential for assimilatory sulfate reduction predicted only for MGIIb MAGs. This family encoded an ATP sulfurylase (*cysD* and *cysN*) that, in conjunction with an APS kinase (*cysC*), could convert sulfate molecules into adenosine 5’-phosphosulfate (APS), followed by the generation of phosphoadenosine phosphosulfate (PAPS), a sulfate source for sulfotransferases involved in the generation of sulfated compounds (Fig. 5). Interestingly, all MGIIa MAGs lacked the ATP sulfurylase subunits and the APS kinase. This finding was consistent for the MGIIa genera L1 (0/40) and M (0/26), but in sharp contrast to MGIIb genera where these genes were prevalent. Known sulfate transporters (e.g., SulT, SulP, CysP, and CysZ) were not detected in MGIIa Helgoland MAGs [62, 63]. These results suggest there may be alternative pathways for sulfur assimilation or incorporation of reduced sulfur-containing organic compounds.

### Peptidases and membrane transport proteins in MGII MAGs

Previous studies proposed a role in organic matter degradation for MGII populations, partly due to the presence of peptidases and several transporters in their genomes [10–12, 58]. Peptidase prediction according to MEROPS classification [64] resulted in 66 sub-families detected in recovered MAGs, comprising, on average, 4.8 and 5.7% of the predicted coding sequences of MGIIa and MGIIb genomes respectively. Families C (cysteine), M (metallo), and S (serine) were among the most abundant. Extracellular peptidases were detected in MGII MAGs using a subcellular localization analysis based on amino acid sequences. For instance, all MGII MAGs encoded a predicted number of one to four copies of the extracellular subtilisin S8, likely involved in degradation of external peptides. Other peptidases are specific to the different MGII clades and might contribute to niche differentiation by targeting specific peptides. Furthermore, M28D and M14A were among the extracellular peptidases most commonly detected in MGIIa and MGIIb Helgoland MAGs (Table S5).

**Fig. 6:**
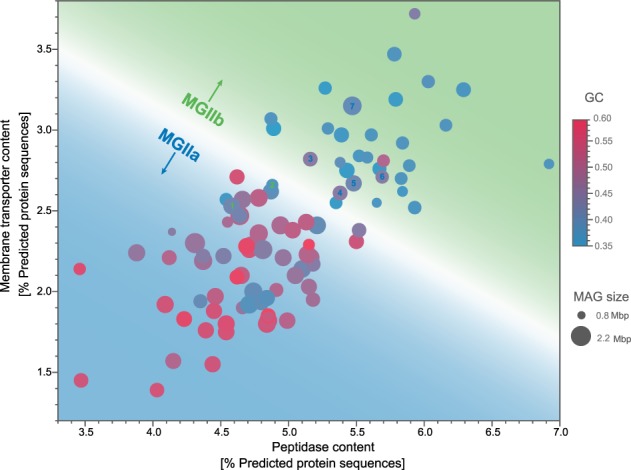
Membrane transporter and peptidase contents in MGII MAGs. The membrane transporter and peptidase contents were determined in recovered MGII MAGs from Helgoland and others recovered from elsewhere around the world [11, 12]. All displayed MAGs belong to the O2, O3, L1, and M genera (showed in Fig. 1b). Highlighted MAGs that deviate from the differential clustering are TMED129 [1], SAT116 [2], 67673 [3], 69590 [4], 67694 [5], UBA111 [6], and RS406 [7]

Membrane transporters in MGII genomes were mostly classified as alpha-type channels and P-P bond hydrolysis, decarboxylation, and oxido-reduction-driven transporters (primary active transporters) according to the TCDB database (Table S6). Most of the detected proteins were potentially involved in the transport of inorganic nutrients (e.g., phosphate) and organic substrates (e.g., amino acids). Nonetheless, the searches were inconclusive in identifying the exact type of transported molecules, in agreement with previous findings [58]. Interestingly, MGIIb MAGs encoded, on average, ~34% more membrane transporters compared to MGIIa genomes (Fig. 6). Thus, MGIIb MAGs encoded, in general, a higher fraction of peptidases and membrane transporters compared to MGIIa. Exceptions to this trend were seven MAGs for which their peptidase and membrane protein contents resembled MAGs belonging to the opposite group (Fig. 6).

### Rhodopsins in MGII MAGs

The presence of single rhodopsin genes in the majority of the MGII MAGs retrieved in this study suggested a photoheterotrophic metabolism (see Figs. S10 and S11 for phylogenetic and sequence analyses). The genetic context surrounding the rhodopsin genes were almost completely syntenic among MGIIa MAGs and also with published MGII genomes (Fig. S12). In MGIIa MAGs, this genomic region was uniformly composed of homologs for the geranylgeranylglyceryl phosphate synthase, a homolog of an ABC-type transporter, genes involved in the metabolism of purines (*purQ* and *purL*), and *uvrA* endonuclease. In contrast, the genetic context of rhodopsin genes in MGIIb MAGs was more variable. For instance, a copy of the rRNA large subunit methyltransferase E (*rlmE*) preceded the rhodopsin gene in three of the recovered MGIIb MAGs. However, MGIIb_c11 cluster encoded a copy of the pyridoxamine 5’-phosphate oxidase gene, similar to the genetic context observed in *Ca*. Thalassoarchaea euryarchaeote reported earlier [14].

## Discussion

### Dynamics and diversity of MGII North Sea populations

Metagenomics, oligotyping of 16S rRNA gene sequences, and microscopy consistently identified MGIIa (*Ca*. Poseidoniaceae) as summer and MGIIb (*Ca*. Thalassarchaeaceae) as winter clades. This is consistent with earlier observations in the Mediterranean Sea [15, 16], off the coast of California [17], and MGII populations recovered at a global scale [11]. Also, sharp increases in MGIIa abundance reported here of up to 23% of the microbial community, have been previously observed after phytoplankton blooms [17, 19] or as sporadic high abundances detected during extended sampling periods [3, 15, 18]. In fact, the high relative *Euryarchaeota* cell counts reported here, reaching up to 1.7 × 10^5^ cells ml^−1^, agreed with earlier reports describing 1–2 × 10^5^ cells ml^−1^ in spring and summer seasons of coastal areas of the North Sea [18]. Our microscopy and oligotyping approaches also agreed with a higher year-round frequency of MGIIa cells in the smaller size fraction. Thus, we hypothesize that their small average cell sizes (~0.7 µm diameter) might provide protection against predation by protozoa in the picoplankton, which is often size selective for cells > 1 µm diameter [65] and could drive the short periods of high MGIIa abundance at the start of the summer.

During the 2009–2012 sampling period, ANIr values, a proxy for quantifying intra-population sequence diversity, were remarkably high for all MGII MAGs (but also in coastal metagenomes from the TARA ocean expedition, see Fig. S13). Previous analyses have proposed that populations showing higher intra-population sequence diversity might represent differentiated/distinct sub-populations characterized by large population sizes and longer evolutionary time since the last intra-population diversity sweep [43]. This previous study has also indicated increased persistence over time for populations showing higher intra-population sequence diversity (i.e., a positive correlation between higher intra-population diversity and increased detection in a series of samples). In contrast, lower intra-population diversity (i.e., more clonal populations) could represent populations that had undergone a recent intra-population diversity sweep event [66]. Our results highlight that almost identical (clonal) MGIIb populations recurred every year in a predictable manner. Thus, we hypothesize that MGIIb populations could be subjected to high selective pressures that promote a particular genetic composition during winter seasons. Their streamlined genomes might also limit the incorporation of new genetic variations (assuming most genetic content has become essential). In contrast, the higher variance in the distribution of ANIr values observed for MGIIa MAGs, in particular to MGIIa_c6 and MGIIa_c5, revealed a higher intra-population diversity for the more persistent MGIIa populations. We hypothesize that MGIIa populations might undergo a higher frequency of selective sweeps compared to MGIIb, potentially due to stronger selection during the summer period when the predation rate is high. Alternatively, stochastic priming events of closely related MGIIa genotypes could emerge during spring and dominate the population throughout the season. The isolation of individual members of the MGII populations and high-resolution population data (e.g., metagenomes and single-cell data) over time would be necessary in order to distinguish between these two distinct scenarios.

### Niche differentiation and metabolic potential of MGII populations of the North Sea

The potential participation of MGII archaea in marine biogeochemical processes has recently been recognized as the result of several studies using culture-independent approaches [10–12, 14]. Our analyses corroborate the suggested photoheterotrophic lifestyle involving protein and lipid degradation for both MGII clades, yet we also corroborated significant differences between sympatric clades MGIIa and MGIIb. The most evident of these features was the smaller average genome size of the winter MGIIb clade where MAGs had an average 1.4 Mbp, significantly smaller than those of MGIIa (~1.9 Mbp). A trend that microorganisms dwelling in substrate-poor settings have smaller genomes has also been proposed [67]. The summer-clade MGIIa MAGs had additional genes such as those encoding for the synthesis of phosphoribosyl pyrophosphate (PRPP; nucleotides pathway), a higher number of flagella-related proteins, and specific peptidases were characteristic of MGIIa MAGs (Fig. 5). However, the presence of these features can in itself not explain the success of MGIIa populations since the MGIIb_c11 MAG encoding some of them (e.g., reduced set of flagella proteins) remained below detection limits in spring and summer. MGIIb_c11 belongs to a genus (O3) previously predicted among the few groups within MGIIb encoding flagellar proteins [11, 12]. In agreement with previous findings [11], no known chemotaxis signal transduction systems were detected, yet the ability to swim towards particles could be an advantage in a post-bloom summer situation.

MGII *Euryarchaeota* lack the genetic resources for the oxidation of small carbon molecules or a large set of hydrolases for the degradation of polysaccharides. High numbers of glycoside hydrolases are characteristic for bacterioplankton bloom populations co-occurring with algal blooms [23, 25]. Previous analyses of MAGs recovered at a global scale have also observed a reduced set of GHs in MGIIa clades and an overall lack of these proteins in MGIIb [12]. We found only one GH (GH1) in Helgoland MGIIa MAGs (clades M and L1) but other GHs reported for members of the M genus (e.g., GH13, GH57, and GH77) were not detected in Helgoland MGIIa MAGs. The low number of detected GHs is likely insufficient for effective degradation of complex glycans and we therefore hypothesize that grazing-resistant MGII use organic matter, including peptides, left in the aftermath of spring algal blooms.

Our data suggests that MGIIb populations are well adapted to conditions at the end of the winter, when most organic substrates have been remineralized [25]. One major advantage they have is a large number of transporters in their genomes [11, 12], which may allow them to better recover what limited organic substrate is available. Although energetically costly, the capacity for sulfate assimilation exclusively found in MGIIb MAGs likely provides a competitive advantage over MGIIa in late winter when reduced sulfur compounds such as DMS, DMSO, and DMSP will likely be at minimum [68]. In contrast, in early summer the massive release of organic matter from phytoplankton during spring blooms in Helgoland [23, 25] will have replenished the pool of readily available reduced sulfur compounds. Therefore, MGIIa populations can forego the sulfate assimilation pathway, similar to other microorganisms dominating the summer season such as SAR11 [69, 70].

The inferred metabolic reconstruction from recovered MAGs highlights the metabolism of major substrates such as fatty acids and proteins. Compared to MGIIa, a higher content of peptidases and membrane transporters were characteristic of MGIIb MAGs from the German Bight and those recovered from elsewhere around the world [11, 12], suggesting that both features significantly contribute to niche differentiation between marine MGII populations (Fig. 6). In addition, extracellular peptidases detected in MGII populations from global surface water analyses [12] and the deep-sea [7] were also detected in MGII MAGs. Nonetheless, only a fraction of them (see Table S5) were predicted as extracellular peptidases using a protein localization prediction algorithm [71]. The extracellular peptidases detected in Helgoland MGII populations (e.g., M28D/F, M14A, and S08) have been previously detected in metatranscriptomes of MGII populations from the deep-sea [7], thus suggesting a potential role for MGII in the recycling of more recalcitrant organic matter after spring phytoplankton blooms. It is worth mentioning the limitations when attempting to predict metabolic potential and phenotype from recovered MAGs. In our case, only an average of 46% of all genes in the MGII MAGs can be annotated with a function, underscoring our limited knowledge of Archaea, especially in MGII populations which lack cultured isolates. Clearly, additional efforts are needed to better resolve the metabolism of the ecologically successful and recurrent MGII populations.

Previous reports have suggested the existence of MGII populations associated with detritus particulate organic matter (POM) that could outnumber free-living MGII in oligotrophic environments [13]. In fact, flagellar components have also been suggested to mediate cell adhesion [11, 72]. Nonetheless, our microscopy and oligotyping approaches both suggested that coastal MGII populations at Helgoland are mostly free-living, although we also found them associated with particles, but less frequently throughout the year.

Based on multi-year sampling, our study documents the diversity and ecology of marine *Euryarchaeota* characteristic of the winter and summer seasons of the coastal North Sea in unprecedented detail. Based on high-quality MAGs, 16S rRNA oligotyping of size-fractionated cells, and CARD-FISH, we detected a pronounced niche separation of annually recurrent discrete MGII populations. Future studies should expand the study of MGII to more sites, acknowledging that abundances of the different clades change strongly over the course of the year and are markedly seasonal. A deeper understanding of key aspects such as motility and chemotactic behavior will, however, require the cultivation (or enrichment) of a representative of MGII. To do so would provide considerable insight into what is, at least in the North Sea, a significant microbial catalyst of organic matter remineralization.

## Supplementary information

Supplementary material

Figure S1

Figure S2

Figure S3

Figure S4

Figure S5

Figure S6

Figure S7

Figure S8

Figure S9

Figure S10

Figure S11

Figure S12

Figure S13

Table S1

Table S2

Table S3

Table S4

Table S5

Table S6
