# Supplementary material for: Niche differentiation among annually recurrent coastal Marine Group II Euryarchaeota
Source: ISME J. 2019 Aug 26;13(12):3024–36. doi: 10.1038/s41396-019-0491-z (PMC6864105; doi:10.1038/s41396-019-0491-z)
Supplement: Supplementary file 1 — Supplementary material [file 41396_2019_491_MOESM1_ESM.docx]

# Supplementary Material and Methods

# Catalyzed reporter deposition fluorescence *in situ* hybridization

# For the enumeration of Euryarchaeota, 100 ml of water were fixed with 1% formaldehyde at room temperature for 1 h, filtered onto 47 mm diameter polycarbonate filters (pore size 0.2 µm) with a vacuum of at most 100 mbar. All filters were dried and stored at -20 °C until further analyses. Catalyzed Reporter Deposition Fluorescence in situ hybridization (CARD-FISH) was done as outlined before (1). In brief, cells on filters were embedded in low gelling point agarose and permeabilized with 10 mg ml-1 lysozyme for 1 h at 37°C. After washing with excess deionized water, endogenous peroxidases were inactivated by incubation in 3% hydrogen peroxide solution for 10 min at room temperature and filters were subsequently again washed thoroughly in deionized water. Two probes were used, one for detecting MGII Euryarchaeota (EURY806; 5’-CACAGCGTTTACACCTAG-3’) (2) with 0% formamide, and the newly developed probe specific for MGIIa_c6 (MGIIa_c6-398; 5’- AAATGCACACACTTGGAG-3’), with 10% formamide in the hybridization buffer. The latter probe was used jointly with two helper oligonucleotides (3) (MGIIa_c6-398-h5 5’-TTCCCTCATCACAGTTTCCTG -3’ and MGIIa_c6-398-h3 %’GAGTTCAGAAAAGTGTACATA). Hybridization was done for 2 h and CARD amplification for 45 min at 46°C. After CARD, filters were washed in deionized water and 80% ethanol, stained with 4',6-diamidino-2-phenylindole (DAPI, 1 μg ml^-1^), embedded in antifading reagent (Citifluor-Vectashield) and quantified by epifluorescence microscopy. At least 15 fields of view were manually counted per sample.

# Reconstruction gene phylogenies

The 120 single copy marker proteins described for MGII genomes (4, 5) were searched using HMMER v3.2.1 (6) and models obtained from TIGRFAM (7), AMPHORA2 (8), and Pfam (9). Single copy markers detected in >90% of MAGs were further used for analyses (n=91). Multiple alignments for each protein were generated using ClustalΩ (10) and a concatenated alignment was generated using the ‘Aln.cat.rb’ script (11). A phylogenetic tree was constructed using FastTree (v2.1.10l -gamma -lg) (12) and visualized in iTol (13).

Genes encoding 16S rRNA gene sequences were detected and extracted from MAGs and referential genomes using Barrnap (https://github.com/tseemann/barrnap). Recovered 16S rRNA gene sequences from MAGs (>800 bp) and reference genomes from public databases were aligned using SINA (14). Conserved gaps were removed from the alignment using GapStreeze v.2.1.0 (https://www.hiv.lanl.gov/content/sequence/GAPSTREEZE/gap.html) and ambiguously aligned regions were also removed. The generated alignment was used for the calculation of a maximum-likelihood tree in RAxML using a GTRCAT model and 1,000 bootstraps.

Genes encoding rhodopsins were searched using rhodopsin reference sequences previously reported (15, 16). An alignment of recovered and reference rhodopsin sequences was generated using ClustalΩ and a maximum-likelihood phylogenetic tree was generated in RAxML (PROTGAMMAAUTO and 1,000 bootstraps).

**Comparison of MAG protein sequences**

Orthologous groups of proteins between MGII MAGs were identified as reciprocal best matches in pairwise BLASTp (BLAST+ 2.7.1) (17) searches (identity >40% and query coverage >70%) and using a clustering algorithm as implemented in the “ogs.mcl.rb” script of the enveomics collection (11). Visualization of orthologous groups was done using the UpSetR package (18) implemented for R 3.5.1 (19).

# Gene annotation

Predicted protein sequences from each MAG were annotated using the UniProt (20) database and BLASTp (cut-off: >40 identity % and 70% alignment coverage). In addition, KEGG annotations were obtained from BlastKOALA (archaea profile) (21) and EggNOG 4.5.1 (archaea profile)(22) for each MAG. Peptidases were searched using predicted protein sequences from each MAG and the MEROPS database (23) using BLASTp, selecting for matches with >40% identity and >70% query aligned. Membrane proteins were detected using predicted protein sequences as query and the Transporter Classification Database (TCDB) (24) in BLASTp searches (cut-off:>50% identity and >70% query aligned). Sub-cellular localization of detected peptidases was predicted using PSORTb v3.0.2 (25). Predicted protein sequences encoding carbohydrate-active enzymes (CAZy) were searched using BLASTp and MAG sequences against the dbCAN database v07202017 (26) (identity >40% and query alignment >70%) and the results for glycoside hydrolase (GH) annotations were confirmed using InterProScan (27, 28).

**16S rRNA gene oligotyping**

Sample collection at Helgoland and processing for amplicon analysis has been described previously (29). Briefly, the two fractions corresponding to size ranges of 0.2 µm to 3 µm, and 3 µm to 10 µm were separated via filtration of the surface water samples (*c*. 1 m depth). Amplicons were generated for both fractions via PCR amplification of the V4 region of the 16S rRNA gene. The primers used were 515 F (5′-GTGCCAGCMGCCGCGGTAA-3′) and 806 R (5′-GGACTACHVGGGTWTCTAAT-3′). Amplicons were sequenced using Illumina MiSeq 2 × 250 bp chemistry at the Department of Energy Joint Genome Institute (DOE-JGI, Walnut Creek, CA, USA). Raw sequence data are stored by JGI in the GOLD database under the project IDs Gp0056779 (‘free living’), and Gp0072732 & Gp0072733 (‘attached’). Amplicon data analysis was carried out as previously reported (29), using Minimum Entropy Decomposition (MED) (30). In brief, raw read pairs were merged and quality filtered, discarding pairs without perfect overlaps during merging. Primers were trimmed with cutadapt (31). Minimum entropy decomposition was then run using minimum substantive abundance (-M) of 100, and decomposition of one nucleotide position at a time (-d 1). Representative oligotypes were then classified against the Silva v132 database (32), using the SilvaNGS webserver and setting clustering identity to 1 (i.e. no clustering). All sequences classified as chloroplast, mitochondria, or with no predicted relative, were removed prior to further analysis. Relative abundance of individual oligotypes in the two datasets was then estimated as the proportion of all reads from a sample matching the representative oligotype sequence. Differentially abundant MGII oligotypes were determined with the DESeq2 package (33).

# Supplementary Results

# Visualization and quantification of abundance MGII using FISH

Even though the metagenomes were more limited in spanning the 2011 and 2012 periods, the abundances determined from the visualization of the Euryarchaeota cells agreed with the overall patterns observed in the metagenomes (Fig. 3b). During the major spring blooms of 2011 and 2012 (i.e., beginning of March until mid-May), Euryarchaeota cell counts ranged from 4 x 10^2^ cells ml^-1^ to 5.1 x 10^3^ cells ml^-1^ and 1.3 x 10^2^ cells ml^-1^ to 6 x 10^3^ cells ml^-1^ during 2011 and 2012, respectively. After the end of the annual spring phytoplankton blooms, Euryarchaeota cells increased up to 1.7 x 10^5^ cells ml^-1^ and 1.5x10^5^ cells ml^-1^ in 2011 and 2012, respectively, comprising up to ~14% of the total cells. MGIIa_c6 cells, ranged from 5.3 x 10^2^ cells ml^-1^ and 1.8 x 10^4^ cells ml^-1^ during 2011 but were much more prominent in 2012 reaching up to 7.3 x 10^4^ cells ml^-1^ (Fig. 3b). In all four years investigated, the increase of euryarchaeotal cell numbers in late spring/early summer coincided with an intermediate decline of bacterial counts correlated with the end of the spring bloom (Fig. 3 and Fig. S7a). Interestingly, a linear relationship was observed between the magnitude phytoplankton bloom (when chlorophyll *a* values were above 10% of the year average) and the number of days between the end of the phytoplankton bloom and the highest MGII cell peaks for each year (*r*^2^=0.75) (Fig. S7b).

# Metabolic potential of North Sea Euryarchaeota

There were 371 predicted protein orthologs shared across the eight MGII MAGs while at the family-level 583 and 330 were specific to MGIIa (*Ca.* Poseidoniaceae) and MGIIb (*Ca.* Thallasarchaeaceae), respectively (Fig. S9). These determinations might represent an underestimate due to differences in completeness among MAGs. Nonetheless, metabolic reconstructions indicated a heterotrophic lifestyle as most of the genes necessary to perform glycolysis and the citric acid cycle were detected in all recovered MGII MAGs (Fig. 5). The detected glycolytic genes encode an Embden-Meyerhof-Parnas (EMP) pathway but some intermediate steps were consistently not detected in MGII MAGs in agreement with previous findings (5, 34). For instance, both families lacked an ADP-dependent kinase or ATP dependent hexokinase responsible for glucose activation at the beginning of glycolysis. In addition, at the final step of glycolysis, MGIIb MAGs lacked the pyruvate kinase enzyme necessary for the conversion of phosphoenolpyruvate (PEP) to pyruvate, in agreement with previous findings (16). While no phosphoenolpyruvate synthase and pyruvate phosphate di-kinase homologs were detected in any of the MGII MAGs, the presence of phosphoenolpyruvate carboxykinase and fructose-1,6-bisphosphatase II might support gluconeogenesis starting by the conversion of oxaloacetate to PEP. Even though most of the tricarboxylic acid cycle enzymes were found in our MGII MAGs, all genomes lacked the α-ketoglutarate dehydrogenase enzyme, in agreement with previously described MGII genomes (16). Genes necessary for oxidative phosphorylation were also present in both families (e.g., NADH dehydrogenase, succinate dehydrogenase, cytochrome b, ATP synthase). The enzymes required for the four-step sequence of the beta-oxidation pathway were also recovered from MAGs of MGIIa and MGIIb clades, in agreement with previous findings highlighting the capabilities of this archaeal group to catabolize fatty acids (5, 15, 34, 35). Interestingly, while MGIIa MAGs encode all necessary enzymes for non-oxidative pentose formation, most members of the MGIIb lack the ribose-phosphate pyrophosphokinase gene, necessary for the phosphoribosyl pyrophosphate (PRPP) and *de novo* generation of purine nucleotides and several amino acids.

# Rhodopsins in North Sea Euryarchaeota

A phylogenetic analysis placed the predicted rhodopsin protein sequences in two clades composed of sequences from MGIIa or MGIIb (Fig. S10). All rhodopsins likely have a non-bacterial or eukaryotic origin as they clustered within the previously described rhodopsin clade B (16). The presence of conserved methionine in position 105 (with respect to EBAC31A08 sequence positions) in all rhodopsins of MGII MAGs (Fig. S11), suggested a green-light absorption maximum, characteristic of microorganisms from shallow depths (36, 37). Additionally, conserved residues aspartic-97 and lysine-108 suggest their function as proton pumps, similar to the *Ca.* Thalassoarchaea rhodopsins described earlier (16). Although a copy of the rhodopsin gene was not found in the MGIIa_c10 MAG, it is likely missing due to assembly and binning issues because all of the other MAGs from the c101-group sharing >98% ANI did encode a copy of this gene.

# Global distribution of MGII MAGs

In order to inspect the global distribution patterns of the North Sea Euryarchaeota, we mapped the metagenomes from the TARA ocean expedition to our recovered MGII MAGs. The detected MGII populations represented closely related populations to those found in the North Sea, in particular, the MGIIb. In fact, MGII communities related to MGIIa MAGs were below the detection limit (i.e., <0.01% of the total metagenome), whereas MAGs from MGIIb populations (in particular MGIIb_c7) were ubiquitous in surface (~5 m) water layers across TARA metagenomes (Fig. S13a). These MGIIb populations were often detected close to the coast, but also in open ocean settings (Fig. S13). Interestingly, the abundance of populations closely related to the MGIIb_c7 MAG ranged from 0.02% to 5.3% of the microbial community in the analyzed metagenomes (Fig. S13b). The highest abundance levels for the MGIIb_c7 MAG were in temperate surface water in the Atlantic stations TARA145 (~400 km from the US coast) and TARA152 (~650 Km from the coast of Spain), which represent open-ocean environments, and were obtained in late winter (February/March) (Fig. S13a).

The recruitment of short-reads against the MGIIb_c7 MAG also allowed us to further characterize the dynamics of sequence-discrete and co-occurring populations. In metagenomes obtained from the open ocean (e.g., TARA 149, 150, and 151), most short-reads were mapped with nucleotide identities between 75 and 90%, probably representing distinct but related populations of the same genus (Fig. S13c). On the other hand, for more coastal metagenomes (e.g., TARA 145, 007, and 023), the nucleotide identity values were higher, likely indicating the presence of populations of the same species (Fig. S13c). Additionally, the ANIr values for the more coastal metagenomes ranged from 99.14% to 99.35%, indicating a low intra-population sequence diversity close to coastal areas.

**Supplementary Figures**

**Figure S1**. Average nucleotide identity (ANI) values between Helgoland MGII MAGs. Values ≥ 70% are reported.

**Figure S2**. Phylogenetic reconstruction using extracted 16S rRNA gene sequences from MAGs and reference genomes. Branch support values between 85-100% are represented by dots.

# Figure S3. Phylogenetic reconstructions of MGII MAGs. Phylogenetic trees using conserved markers from a collection of 120 single copy gene markers (A) and 15 syntenic genes (B). Tree B represents the expanded version of Figure 1.b. Tree A incorporates all MGII MAGs in Supplementary Table 1. Clades representing previously described genera (5) are highlighted in different colors. Bootstrap values are denoted by black dots scaled from 75% to 100%. The same outgroups were used in both trees: Marine Group III euryarchaeote CG-Epi1 (ASM187534v1), Haloarcula sp. CBA1115 (ASM82783v1), Pyrobaculum sp. WP30 (ASM118927v1), Marine Group I thaumarchaeote SCGC AAA799-B03 (ASM74678v1), *Candidatus* Nitrososphaera gargensis Ga9.2 (ASM30315v1), Nitrososphaera viennensis EN76 (ASM69878v1).

**Figure S4**. Average amino acid identity (AAI) values among representative Helgoland MGII MAGs.

**Figure S5**. Read recruitment plot for MGIIa_c6 MAG using metagenomic reads from metagenome COGITO 998_met_07 (PRJNA266679) obtained the 26^th^ of May 2011. The top left panel shows the average sequencing depth across the MGIIa_c6 MAG (logarithmic scale). The top right panel shows a sequencing depth histogram and detected peaks above sequence identity 98% (sequence discontinuity region). The bottom left panel shows the reads recruited along the MGIIa_c6 MAG (x-axis) and identity (y-axis). The bottom right panel shows a density histogram of the mapping reads in gray and a smoothed spline in black (logarithmic scale). Dark and light blue lines correspond to reads matching above and below 98% identity threshold.

**Figure S6**. Detection of MGIIa_c6 cells in Helgoland during summer**.** Cells shown were obtained from a sample from August 16^th^ of 2012 (summer). **A** Nucleic acids from all cells in the samples are shown in blue (DAPI, 405 nm laser excitation, detection window 420-460 nm). **B** MGII_c6 cells are shown in green and were detected by CARD-FISH using the probe developed in this study (fluorescently labeled tyramide Alexafluor488, 488 nm laser excitation, detection window 495-550 nm). The scale bar represents 1 nm. Imaging done on a Zeiss LSM 780 confocal laser scanning microscope with Airyscan super-resolution technique, using a 63x / 1.4 Plan-Apochromat objective lens, and the ZEN software package for AirScan processing (Carl Zeiss, Jena, Germany).

**Figure S7**. CARD-FISH cell counts for 2010-2012 at Helgoland. **A** Total cell counts (TCC) (left y-axis) vs EURY806 (right y-axis) counts. **B** TCC vs EURY806 (left y-axis) vs. chlorophyll *a* levels (right y-axis). Blue area shows the days between the start and the end of the phytoplankton bloom when chloroyphyll *a* values were above 10% of the year average and the highest MGII cell peaks for each year.

**Figure S8**. Abundance of MGII oligotypes in surface waters. The relative read abundance for selected MGII oligotypes in the 3-10 µm fraction and 0.2-3 µm fractions are shown. MGIIa and MGIIb oligotypes are depicted in blue and green colors, respectively.

**Figure S9**. Orthologous groups of protein sequences among MGII MAGs from Helgoland. Plot shows above ten protein sequences shared among MGII MAGs.

**Figure S10.** Phylogenetic tree of rhodopsins detected in MGII MAGs. Given that a copy of the rhodopsin gene was absent in the MGIIa_c10 MAG, a predicted protein sequence of the MAG MGIIa_c10_3 (genomes shared >98% ANI) was used.

**Figure S11.** Alignment of the protein sequences of detected rhodopsins in MAGs and known references. Selected sequences used for the generation of the phylogenetic tree (Figure S10) were used.

**Figure S12.** Genomic contexts of rhodopsins in MAGs.

**Figure S13**. **A** Global distribution of MGIIb populations based on Tara Ocean datasets. MGIIb_c7 populations were mostly detected close to coastal areas within the 25-50 latitude range and in waters having an average temperature of 18.1°C. Panels **b**, **c**, and **d** show the abundance, distribution, and ANIr values for the recruitment of short-reads against the MGIIb_c7 MAG. TARA metagenomes used for determining MGII abundances: TARA133 (ERR599052), TARA137 (ERR598989), TARA138, TARA140 (ERR599162), TARA102 (ERR598978), TARA109 (ERR599118), TARA142 (ERR599136), TARA145 (ERR598983), TARA146 (ERR598968), TARA148 (ERR599123), TARA149 (ERR598963), TARA150 (ERR599170), TARA151 (ERR598976), TARA152 (ERR599078), TARA004 (ERR598955,ERR599003), TARA007 (ERR315857), TARA009 (ERR594288), TARA018 (ERR594358), TARA025 (ERR598951), TARA022 (ERR594378), TARA030 (ERR315862), TARA031 (ERR598969), TARA032 (ERR599041), TARA033 (ERR599049), TARA034 (ERR594328), TARA036 (ERR594334), TARA048 (ERR599138), TARA056 (ERR599057), TARA064 (ERR598970).

**Supplementary Tables**

**Table S1**. Extended information for all Helgoland MAGs used in this study. Names in bold correspond to the representative MAG used in this study.

**Table S2.** Selected representative MGII MAGs after de-replication (ANI ≥ 99%). MGII MAGs previously reported (5, 34) were de-replicated using ANI ≥ 99% and selected representatives were used for analyses (see more details about de-replication in Methods).

**Table S3. Statistics for MGII** MAGs used for the phylogenetic reconstructions. Table contains statistics for de-replicated MGII MAGs obtained from Rinke et al. and Tully.

**Table S4**. Annotation of predicted protein sequences in *Euryarchaeota* MAGs. Note the zero (null) values might also reflect limitation of the homology search and not necessarily absence of a particular genetic function.

**Table S5**. Summary of detected peptidases. The MEROPS sequence collection was used as a database in BLASTp searches using predicted protein sequences from each MGII MAG. Predicted sub-cellular localization of detected peptidases by PSORTb are shown as “M” for membrane, “E” for external, and “E/M/-” for either external, membrane or unresolved.

**Table S6**. Summary of detected membrane transport proteins. The Transporter Classification Database (TCDB) was used for BLASTp searches using predicted protein sequences from MGII MAGs.

**Supplementary References**

1. Thiele S, Fuchs B, Amann R. Identification of microorganisms using the ribosomal RNA approach and fluorescence in situ hybridization. In: Wilderer PA, editor. Treatise on Water Science: Elsevier Science; 2011. p. 171–89.

2. Teira E, Reinthaler T, Pernthaler A, Pernthaler J, Herndl GJ. Combining catalyzed reporter deposition-fluorescence in situ hybridization and microautoradiography to detect substrate utilization by bacteria and Archaea in the deep ocean. Appl Environ Microbiol. 2004;70(7):4411-4.

3. Fuchs BM, Glockner FO, Wulf J, Amann R. Unlabeled helper oligonucleotides increase the in situ accessibility to 16S rRNA of fluorescently labeled oligonucleotide probes. Appl Environ Microbiol. 2000;66(8):3603-7.

4. Parks DH, Chuvochina M, Waite DW, Rinke C, Skarshewski A, Chaumeil PA, et al. A standardized bacterial taxonomy based on genome phylogeny substantially revises the tree of life. Nat Biotechnol. 2018;36(10):996-1004.

5. Rinke C, Rubino F, Messer LF, Youssef N, Parks DH, Chuvochina M, et al. A phylogenomic and ecological analysis of the globally abundant Marine Group II archaea (Ca. Poseidoniales ord. nov.). ISME J. 2019;13(3):663-75.

6. Eddy SR. Accelerated Profile HMM Searches. PLoS Comput Biol. 2011;7(10):e1002195.

7. Selengut JD, Haft DH, Davidsen T, Ganapathy A, Gwinn-Giglio M, Nelson WC, et al. TIGRFAMs and Genome Properties: tools for the assignment of molecular function and biological process in prokaryotic genomes. Nucleic Acids Res. 2007;35(Database issue):D260-4.

8. Wu M, Scott AJ. Phylogenomic analysis of bacterial and archaeal sequences with AMPHORA2. Bioinformatics. 2012;28(7):1033-4.

9. El-Gebali S, Mistry J, Bateman A, Eddy SR, Luciani A, Potter SC, et al. The Pfam protein families database in 2019. Nucleic Acids Res. 2019;47(D1):D427-D32.

10. Sievers F, Higgins DG. Clustal Omega for making accurate alignments of many protein sequences. Protein Sci. 2018;27(1):135-45.

11. Rodriguez-R LM, Konstantinidis KT. The enveomics collection: a toolbox for specialized analyses of microbial genomes and metagenomes. PeerJ Preprints. 2016.

12. Price MN, Dehal PS, Arkin AP. FastTree 2--approximately maximum-likelihood trees for large alignments. PLoS One. 2010;5(3):e9490.

13. Letunic I, Bork P. Interactive tree of life (iTOL) v3: an online tool for the display and annotation of phylogenetic and other trees. Nucleic Acids Res. 2016;44(W1):W242-5.

14. Pruesse E, Peplies J, Glockner FO. SINA: accurate high-throughput multiple sequence alignment of ribosomal RNA genes. Bioinformatics. 2012;28(14):1823-9.

15. Iverson V, Morris RM, Frazar CD, Berthiaume CT, Morales RL, Armbrust EV. Untangling genomes from metagenomes: revealing an uncultured class of marine Euryarchaeota. Science. 2012;335(6068):587-90.

16. Martin-Cuadrado AB, Garcia-Heredia I, Moltó AG, López-Úbeda R, Kimes N, López-García P, et al. A new class of marine Euryarchaeota group II from the Mediterranean deep chlorophyll maximum. ISME J. 2015;9(7):1619-34.

17. Camacho C, Coulouris G, Avagyan V, Ma N, Papadopoulos J, Bealer K, et al. BLAST+: architecture and applications. BMC Bioinformatics. 2009;10:421.

18. Conway JR, Lex A, Gehlenborg N. UpSetR: an R package for the visualization of intersecting sets and their properties. Bioinformatics. 2017;33(18):2938-40.

19. R Core Team. R: A Language and Environment for Statistical Computing. Vienna, Austria: R Foundation for Statistical Computing; 2018.

20. The UniProt C. UniProt: the universal protein knowledgebase. Nucleic Acids Res. 2017;45(D1):D158-D69.

21. Kanehisa M, Sato Y, Morishima K. BlastKOALA and GhostKOALA: KEGG Tools for Functional Characterization of Genome and Metagenome Sequences. J Mol Biol. 2016;428(4):726-31.

22. Huerta-Cepas J, Szklarczyk D, Forslund K, Cook H, Heller D, Walter MC, et al. eggNOG 4.5: a hierarchical orthology framework with improved functional annotations for eukaryotic, prokaryotic and viral sequences. Nucleic Acids Res. 2016;44(D1):D286-93.

23. Rawlings ND, Barrett AJ, Thomas PD, Huang X, Bateman A, Finn RD. The MEROPS database of proteolytic enzymes, their substrates and inhibitors in 2017 and a comparison with peptidases in the PANTHER database. Nucleic Acids Res. 2018;46(D1):D624-D32.

24. Saier MH, Jr., Reddy VS, Tsu BV, Ahmed MS, Li C, Moreno-Hagelsieb G. The Transporter Classification Database (TCDB): recent advances. Nucleic Acids Res. 2016;44(D1):D372-9.

25. Yu NY, Wagner JR, Laird MR, Melli G, Rey S, Lo R, et al. PSORTb 3.0: improved protein subcellular localization prediction with refined localization subcategories and predictive capabilities for all prokaryotes. Bioinformatics. 2010;26(13):1608-15.

26. Yin Y, Mao X, Yang J, Chen X, Mao F, Xu Y. dbCAN: a web resource for automated carbohydrate-active enzyme annotation. Nucleic Acids Res. 2012;40(Web Server issue):W445-51.

27. Jones P, Binns D, Chang HY, Fraser M, Li W, McAnulla C, et al. InterProScan 5: genome-scale protein function classification. Bioinformatics. 2014;30(9):1236-40.

28. Finn RD, Attwood TK, Babbitt PC, Bateman A, Bork P, Bridge AJ, et al. InterPro in 2017-beyond protein family and domain annotations. Nucleic Acids Res. 2017;45(D1):D190-D9.

29. Chafee M, Fernàndez-Guerra A, Buttigieg PL, Gerdts G, Eren AM, Teeling H, et al. Recurrent patterns of microdiversity in a temperate coastal marine environment. ISME J. 2018;12(1):237-52.

30. Eren AM, Morrison HG, Lescault PJ, Reveillaud J, Vineis JH, Sogin ML. Minimum entropy decomposition: unsupervised oligotyping for sensitive partitioning of high-throughput marker gene sequences. ISME J. 2015;9(4):968-79.

31. Martin M. Cutadapt removes adapter sequences from high-throughput sequencing reads. EMBnet journal. 2011;17(1):pp. 10-2.

32. Quast C, Pruesse E, Yilmaz P, Gerken J, Schweer T, Yarza P, et al. The SILVA ribosomal RNA gene database project: improved data processing and web-based tools. Nucleic Acids Res. 2013;41(Database issue):D590-6.

33. Love MI, Huber W, Anders S. Moderated estimation of fold change and dispersion for RNA-seq data with DESeq2. Genome Biol. 2014;15(12):550.

34. Tully BJ. Metabolic diversity within the globally abundant Marine Group II Euryarchaea offers insight into ecological patterns. Nat Commun. 2019;10(1):271.

35. Zhang CL, Xie W, Martin-Cuadrado AB, Rodriguez-Valera F. Marine Group II Archaea, potentially important players in the global ocean carbon cycle. Front Microbiol. 2015;6:1108.

36. Man D, Wang W, Sabehi G, Aravind L, Post AF, Massana R, et al. Diversification and spectral tuning in marine proteorhodopsins. EMBO J. 2003;22(8):1725-31.

37. Fuhrman JA, Schwalbach MS, Stingl U. Proteorhodopsins: an array of physiological roles? Nat Rev Microbiol. 2008;6(6):488-94.
