# Supplementary figures and images for: Niche differentiation among annually recurrent coastal Marine Group II Euryarchaeota
Source: ISME J. 2019 Aug 26;13(12):3024–36. doi: 10.1038/s41396-019-0491-z (PMC6864105; doi:10.1038/s41396-019-0491-z)

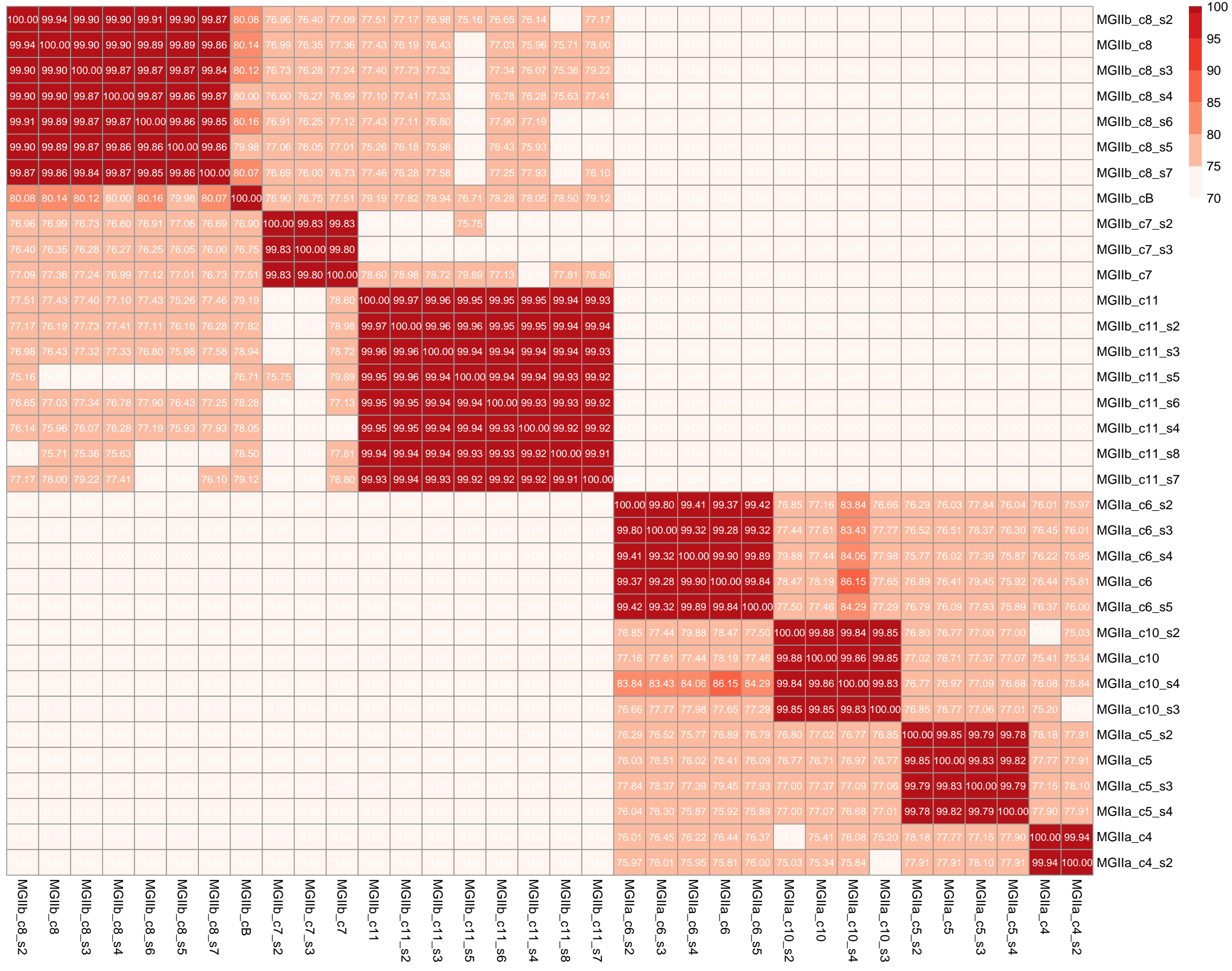

Supplement: Supplementary file 2 — Figure S1 [file 41396_2019_491_MOESM2_ESM.pdf]

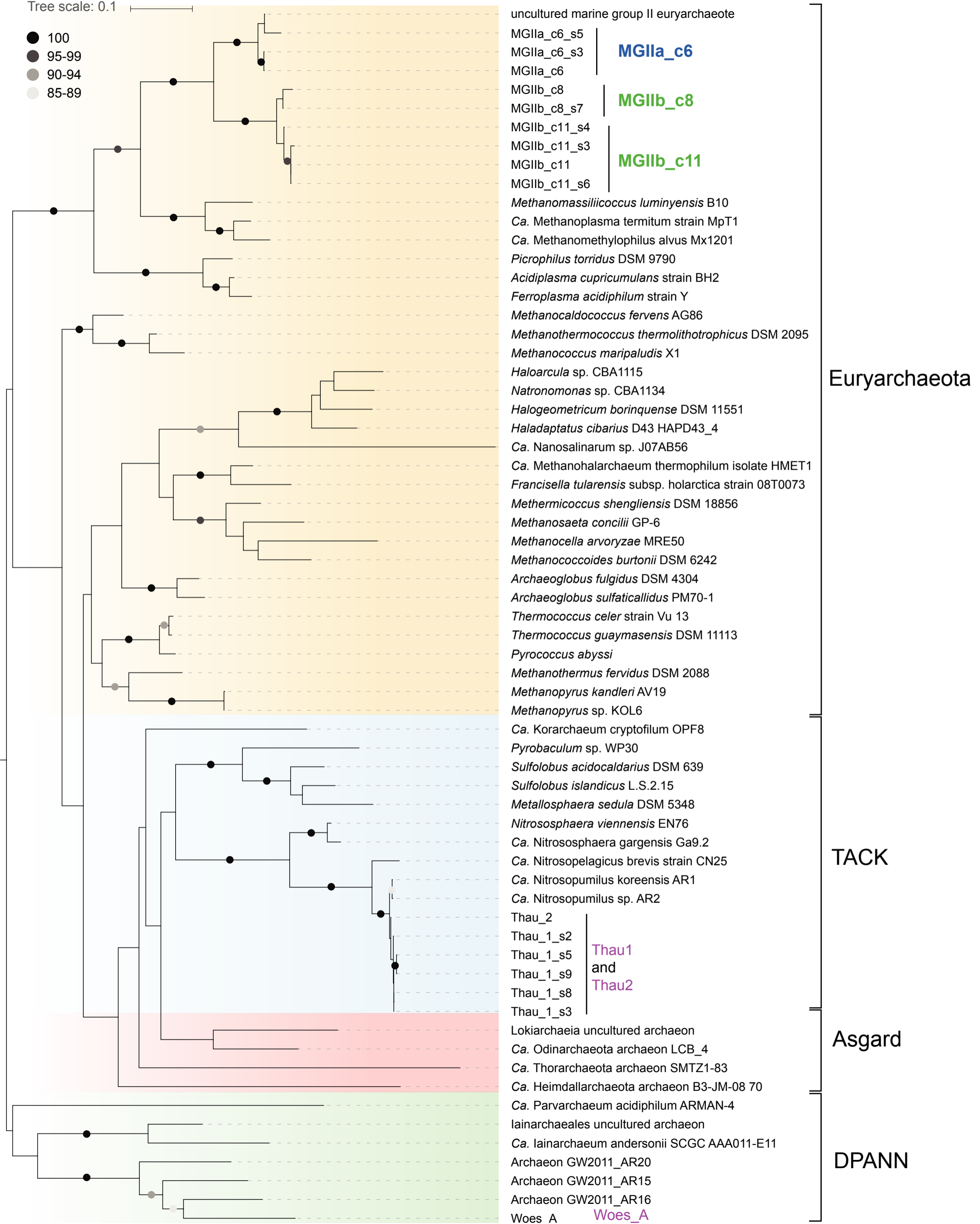

Supplement: Supplementary file 3 — Figure S2 [file 41396_2019_491_MOESM3_ESM.pdf]

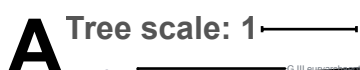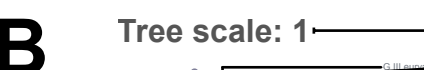

Supplement: Supplementary file 4 — Figure S3 [file 41396_2019_491_MOESM4_ESM.pdf]

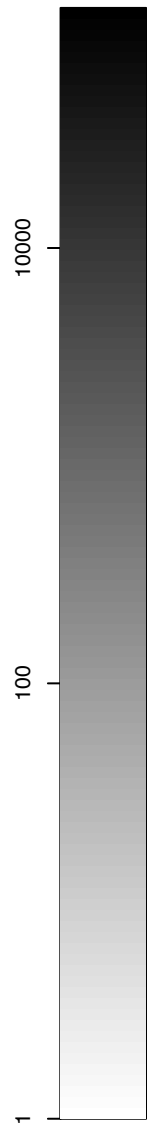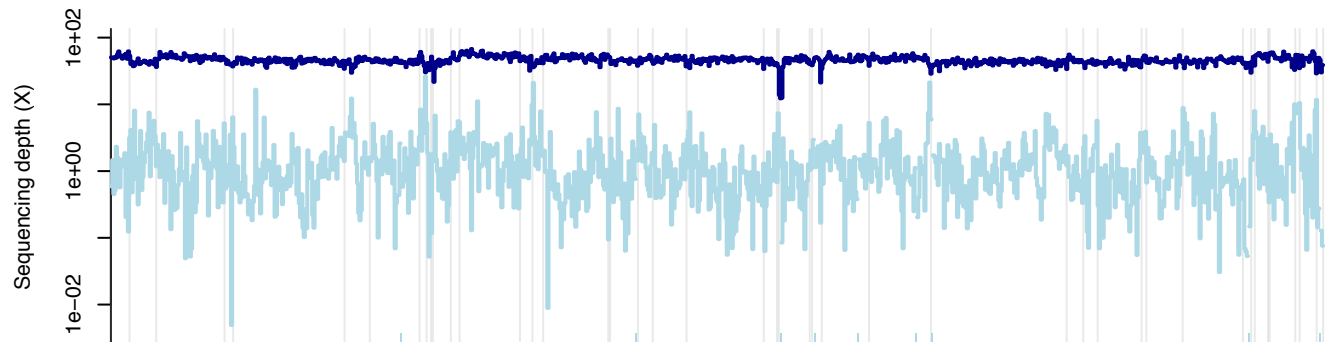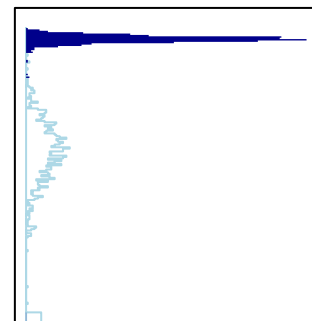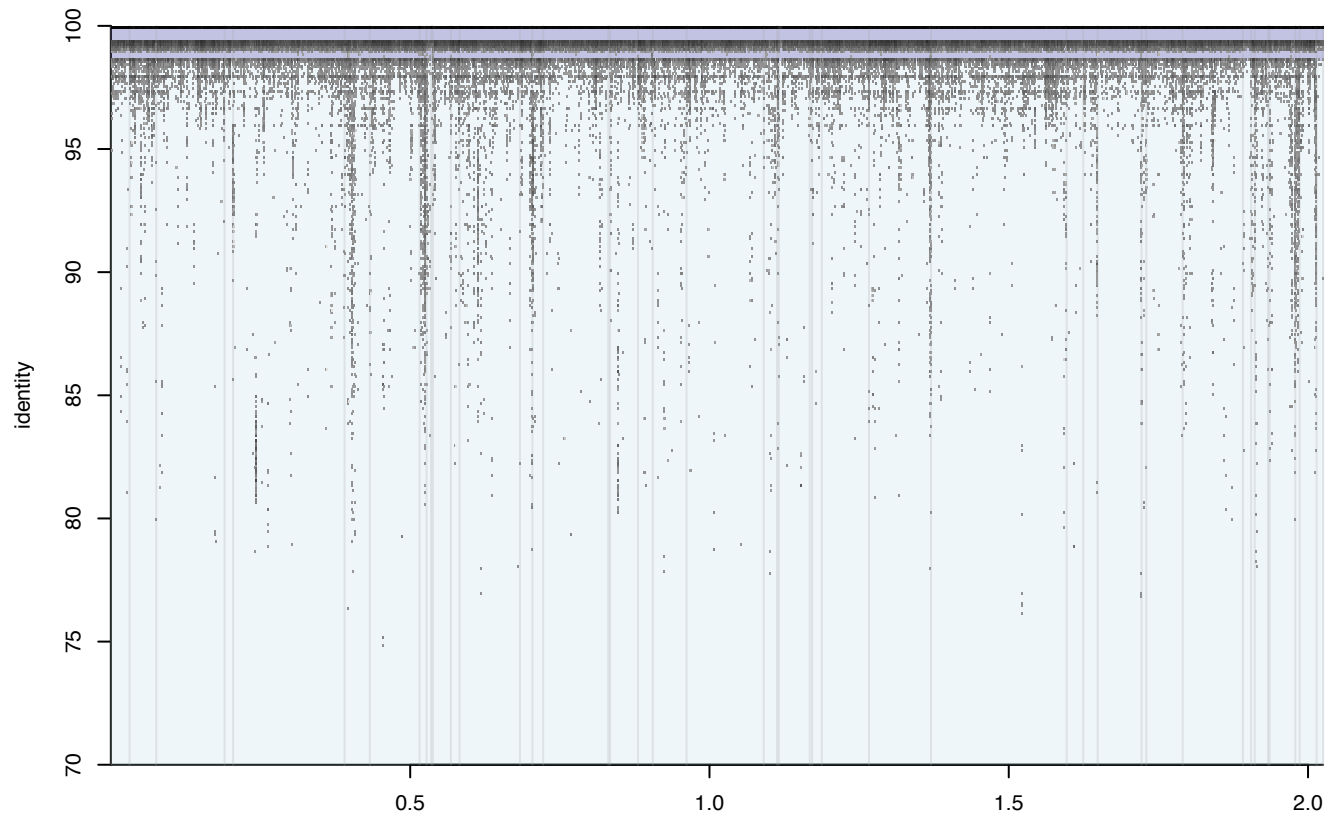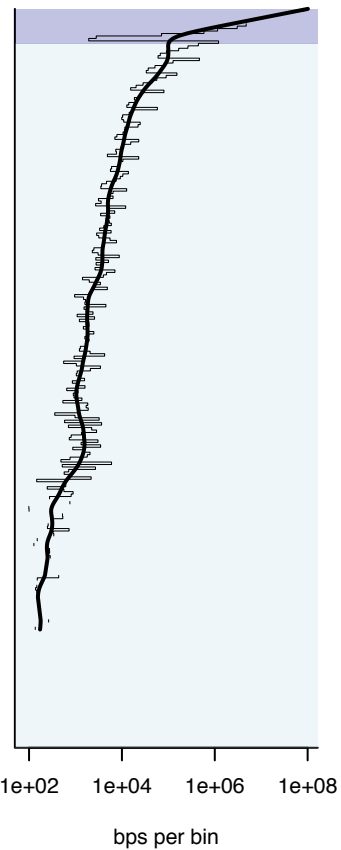

Supplement: Supplementary file 6 — Figure S5 [file 41396_2019_491_MOESM6_ESM.pdf]

**A**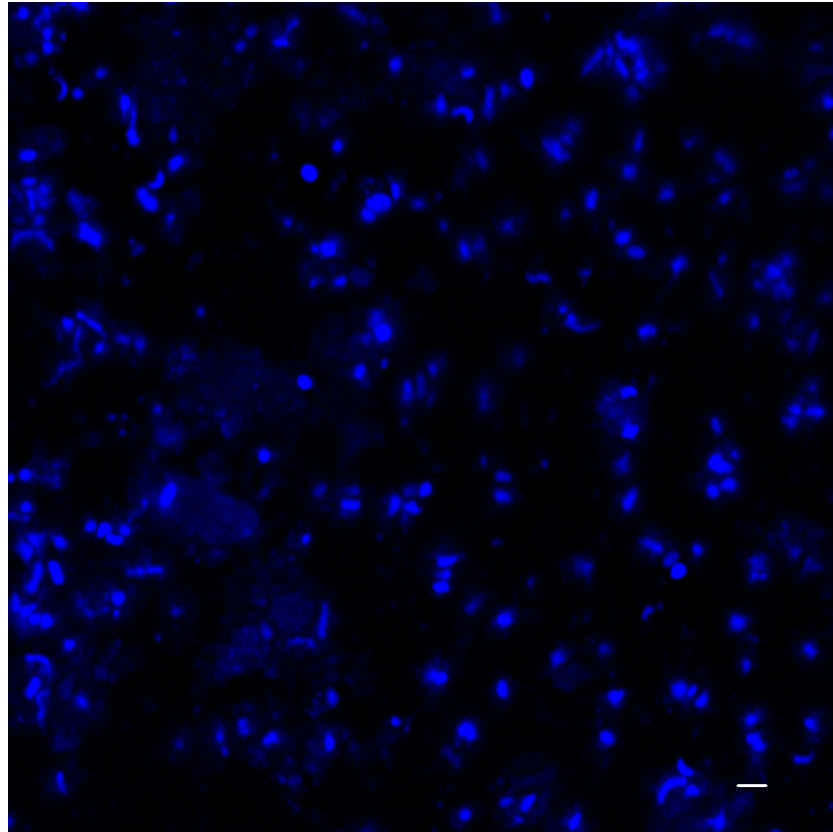**B**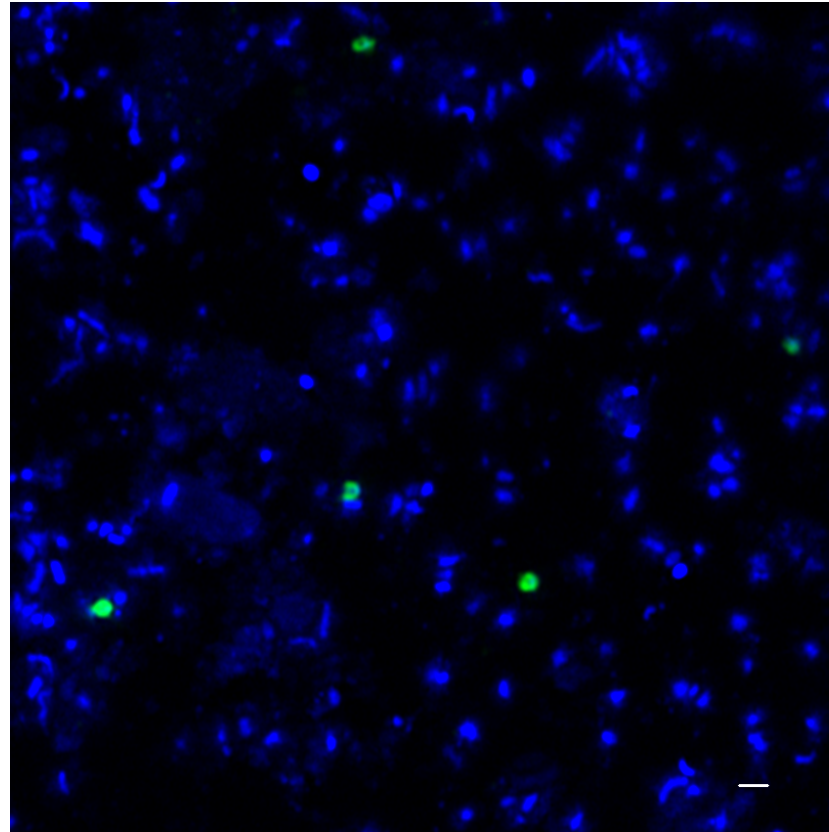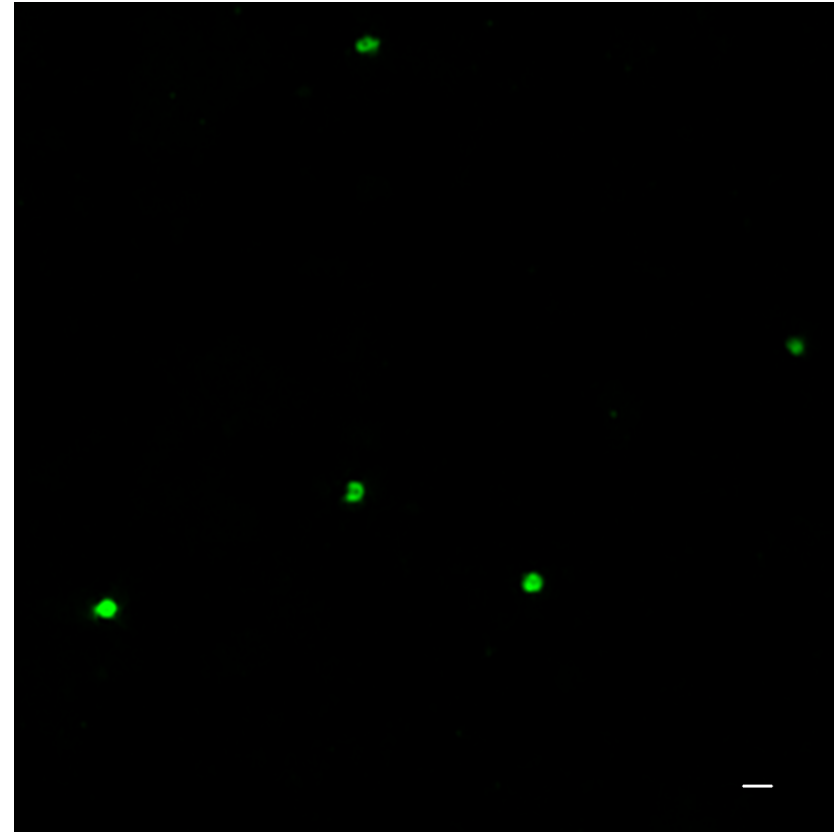

Supplement: Supplementary file 7 — Figure S6 [file 41396_2019_491_MOESM7_ESM.pdf]

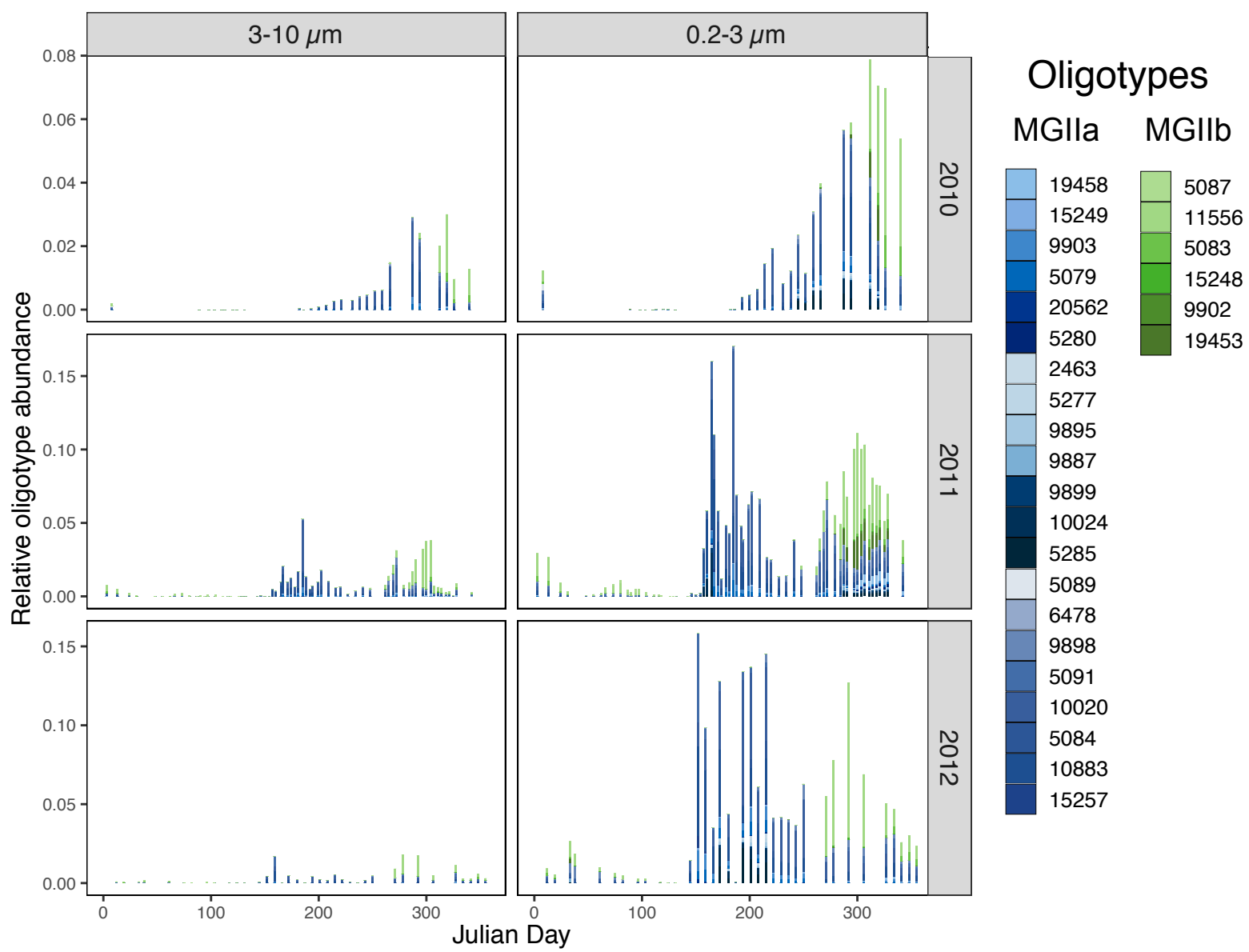

Supplement: Supplementary file 9 — Figure S8 [file 41396_2019_491_MOESM9_ESM.pdf]

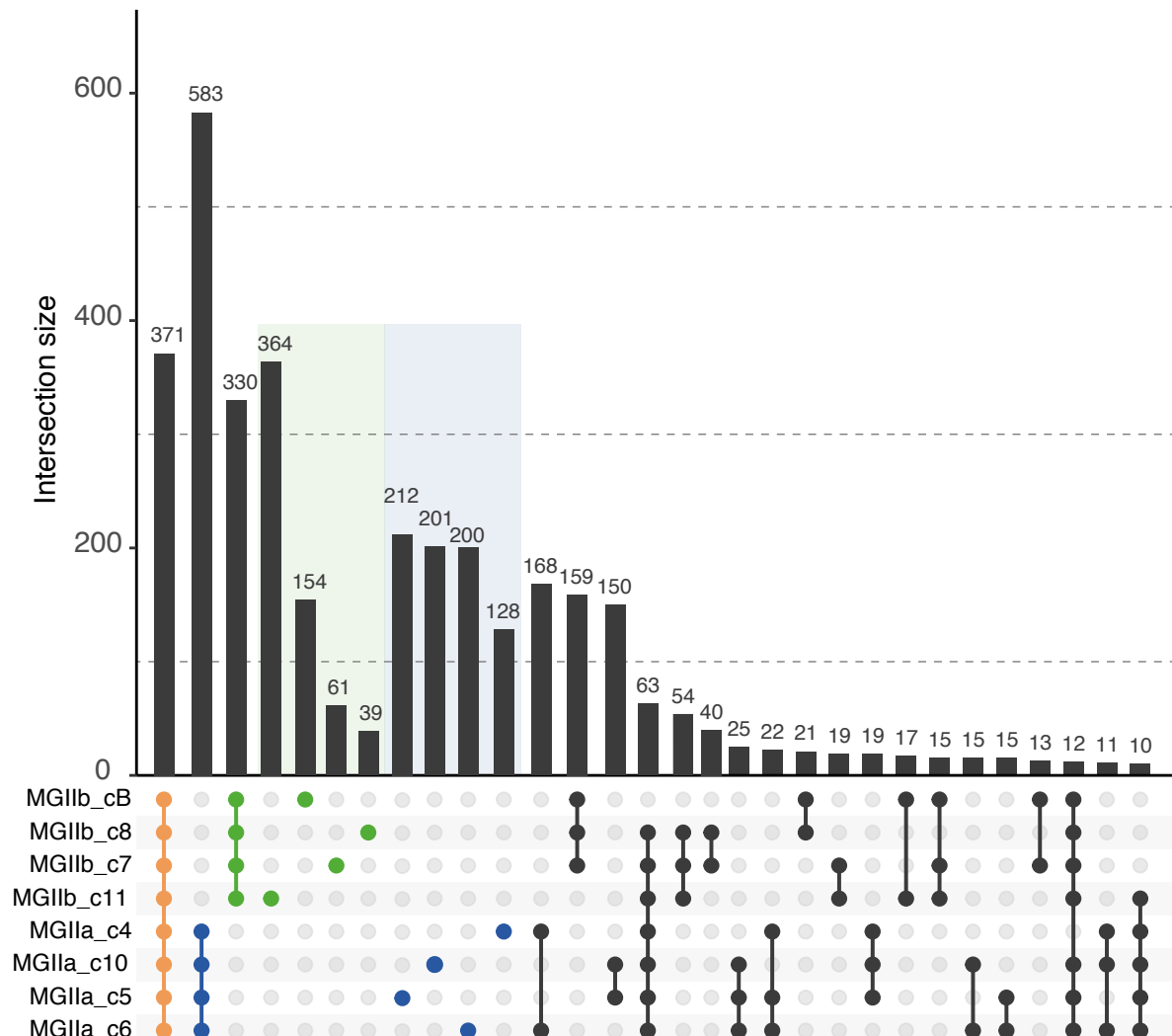

Supplement: Supplementary file 10 — Figure S9 [file 41396_2019_491_MOESM10_ESM.pdf]

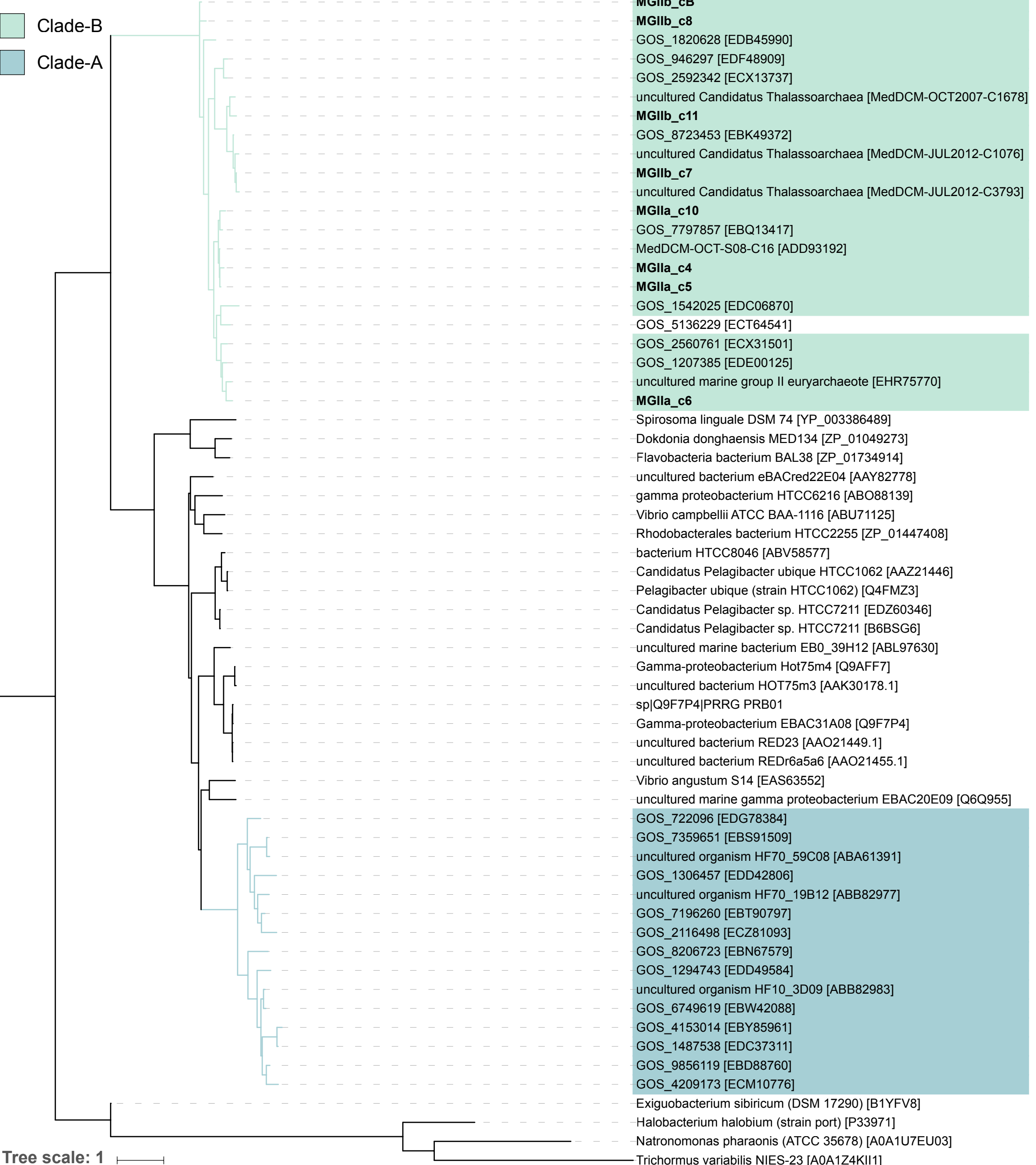

Supplement: Supplementary file 11 — Figure S10 [file 41396_2019_491_MOESM11_ESM.pdf]

100% 80-100% 60-80% < 60%

Similarity

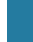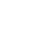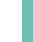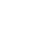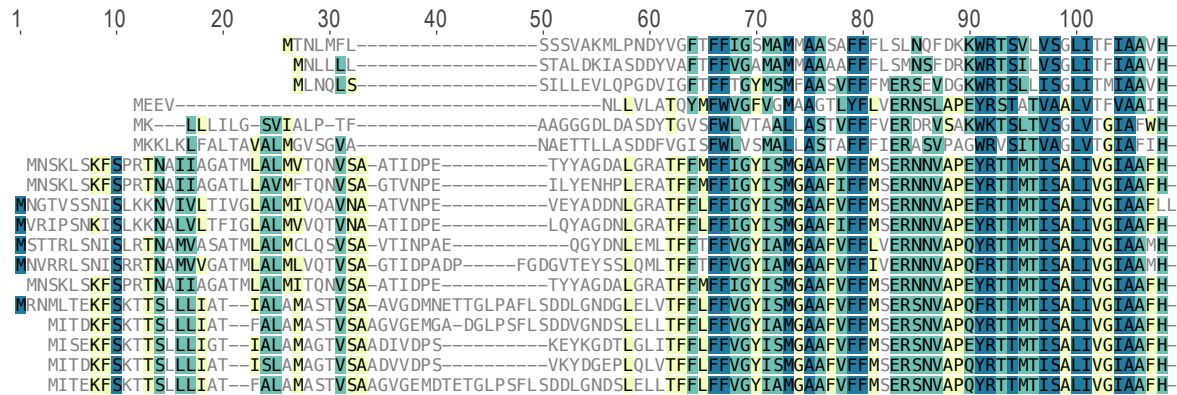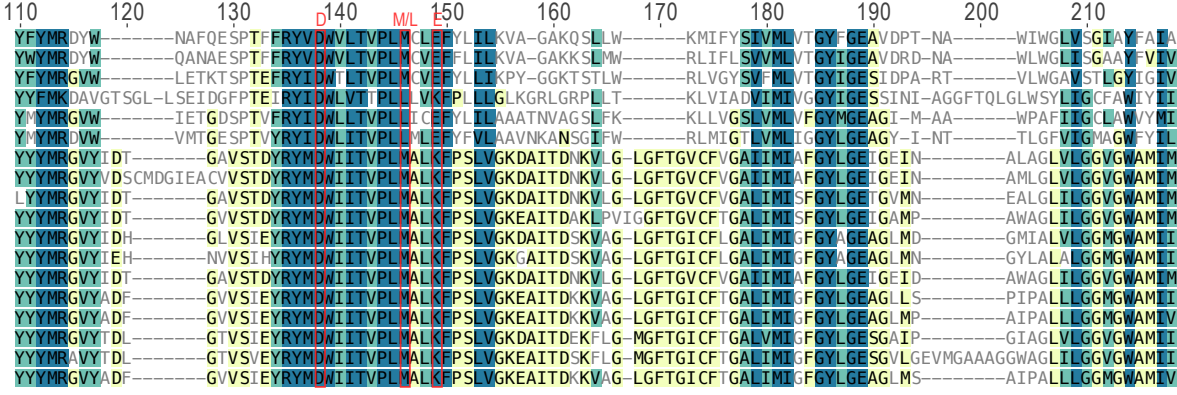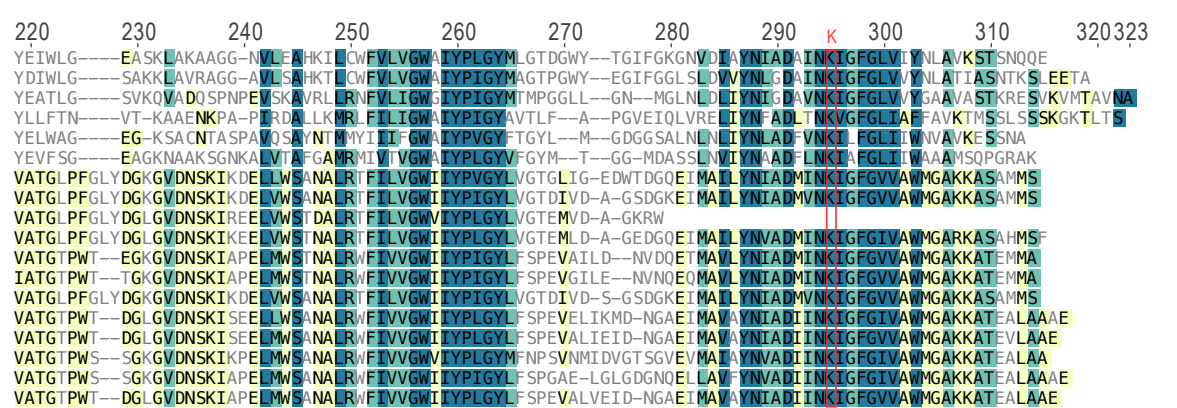

Supplement: Supplementary file 12 — Figure S11 [file 41396_2019_491_MOESM12_ESM.pdf]

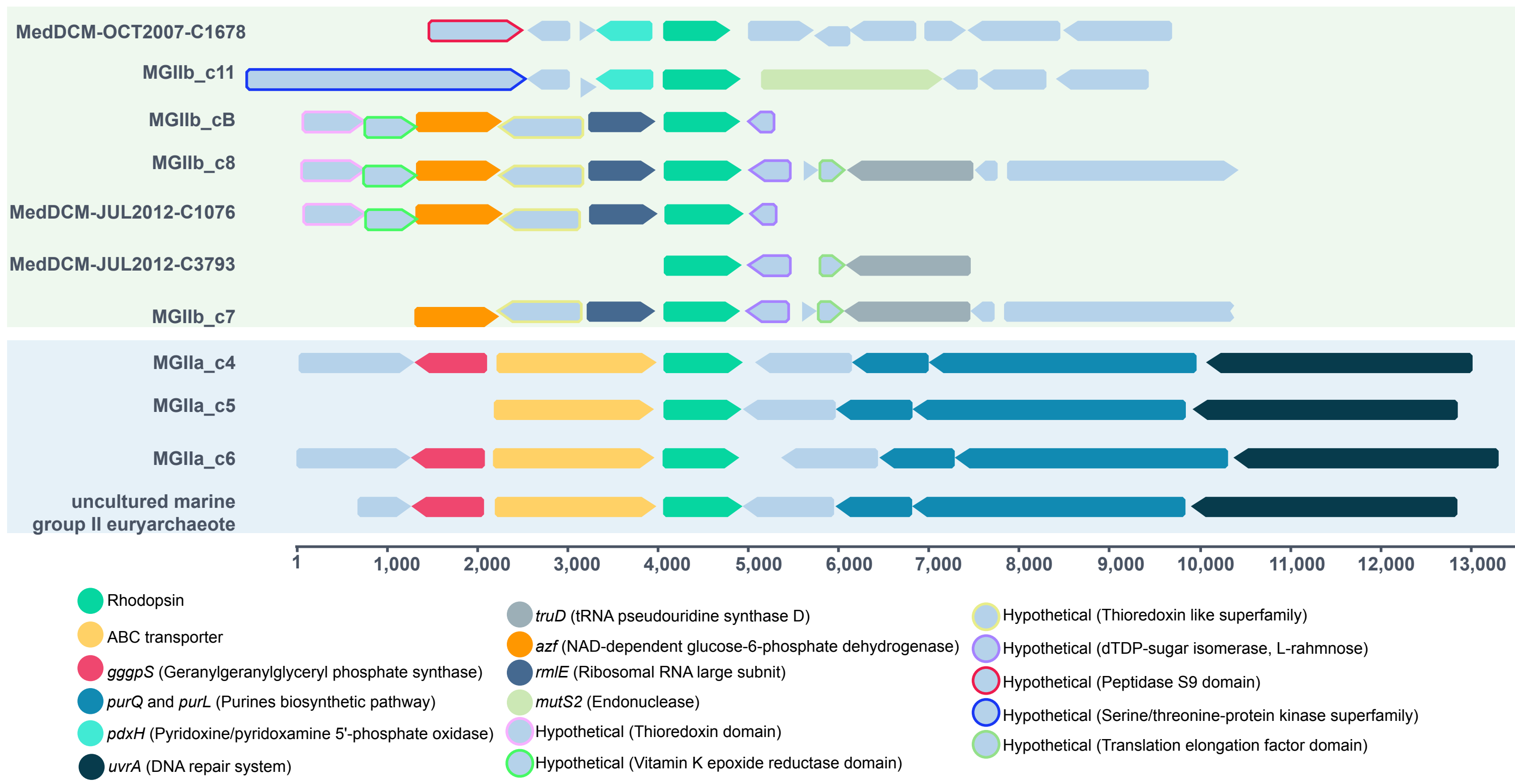

Supplement: Supplementary file 13 — Figure S12 [file 41396_2019_491_MOESM13_ESM.pdf]

**A**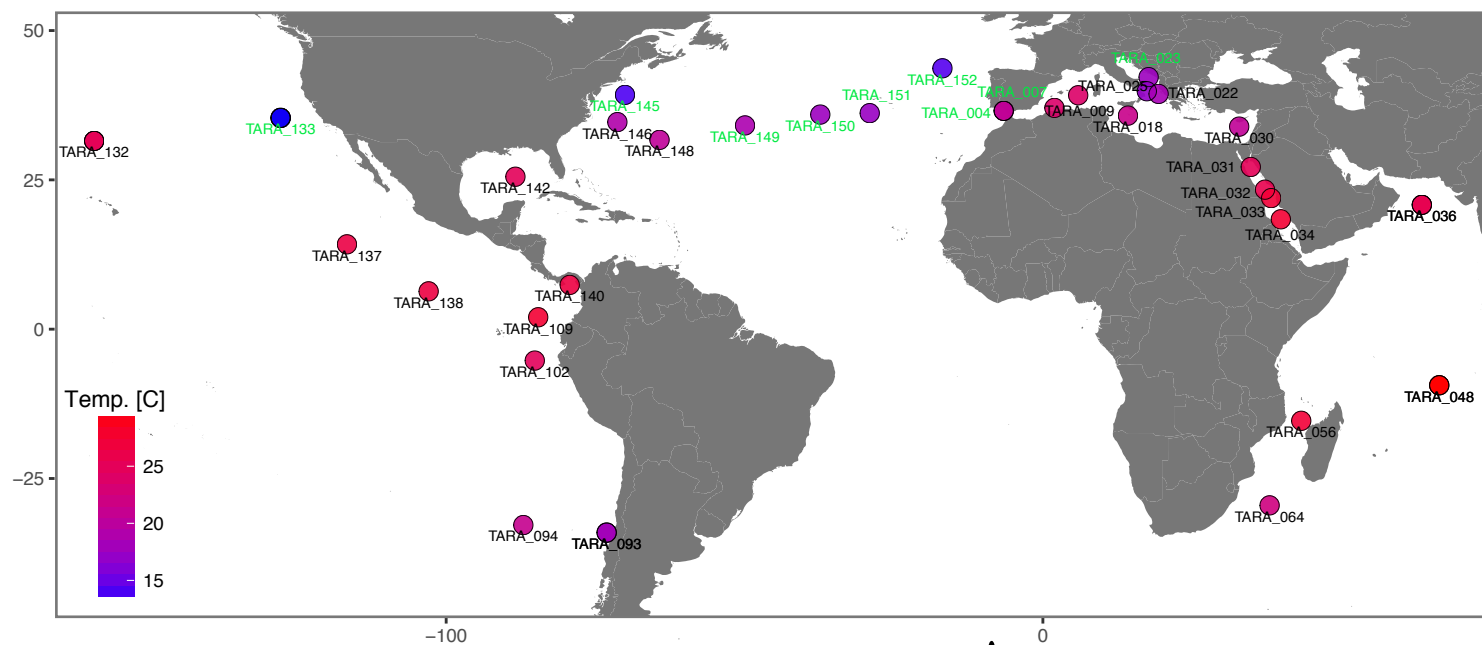**B**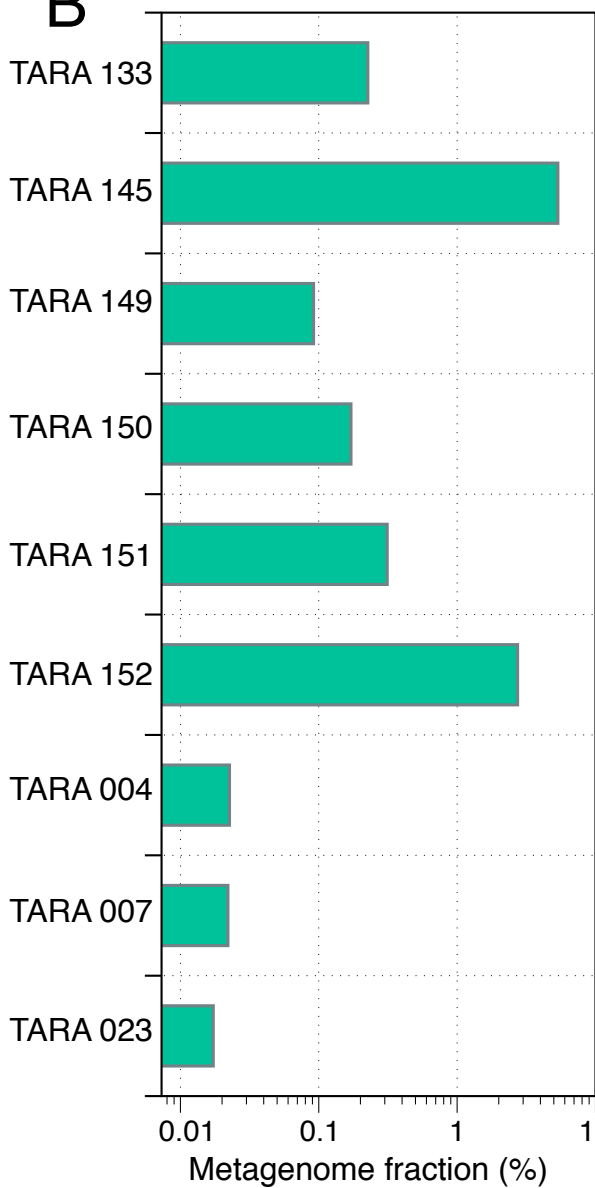**C**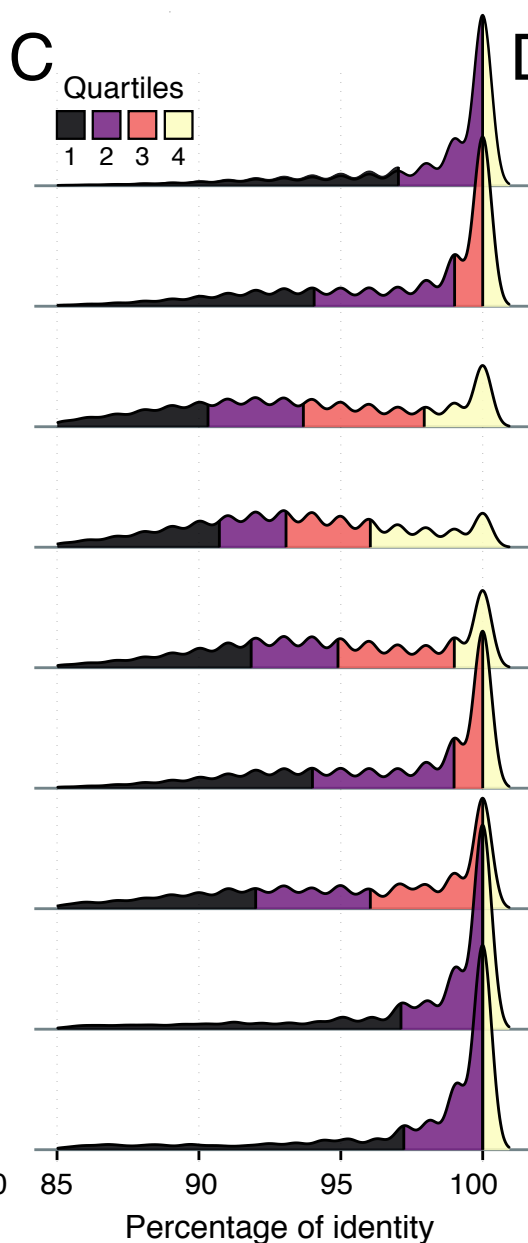**D**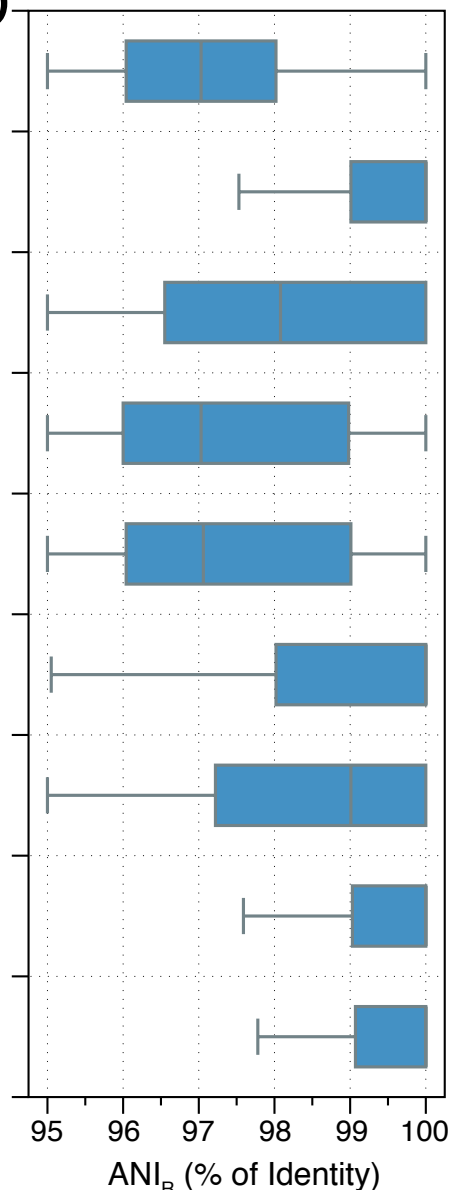

Supplement: Supplementary file 14 — Figure S13 [file 41396_2019_491_MOESM14_ESM.pdf]
