# Supplementary material for: Niche differentiation among annually recurrent coastal Marine Group II Euryarchaeota
Source: ISME J. 2019 Aug 26;13(12):3024–36. doi: 10.1038/s41396-019-0491-z (PMC6864105; doi:10.1038/s41396-019-0491-z)
Supplement: Supplementary file 5 — Figure S4 [file 41396_2019_491_MOESM5_ESM.pdf]

	MGIIb_cB	MGIIb_c8	MGIIb_c7	MGIIb_c11	MGIIa_c5	MGIIa_c10	MGIIa_c4	MGIIa_c6
MGIIb_cB	100	85	80	63	49	49	48	49
MGIIb_c8	85	100	80	63	50	50	49	49
MGIIb_c7	80	80	100	63	49	49	49	49
MGIIb_c11	63	63	63	100	48	48	48	48
MGIIa_c5	49	50	49	48	100	72	64	65
MGIIa_c10	49	50	49	48	72	100	64	65
MGIIa_c4	48	49	49	48	64	64	100	73
MGIIa_c6	49	49	49	48	65	65	73	100
